# Extensive accumulation of misfolded protein aggregates during natural aging and senescence

**DOI:** 10.3389/fnagi.2022.1090109

**Published:** 2023-01-26

**Authors:** Karina Cuanalo-Contreras, Jonathan Schulz, Abhisek Mukherjee, Kyung-Won Park, Enrique Armijo, Claudio Soto

**Affiliations:** ^1^Mitchell Center for Alzheimer’s Disease and Related Brain Disorders, Department of Neurology, McGovern Medical School, University of Texas Health Science Center at Houston, Houston, TX, United States; ^2^Facultad de Medicina, Universidad de los Andes, Santiago, Chile

**Keywords:** protein misfolding, amyloid, prions, aggresomes, senescence, proteostasis, aging

## Abstract

Accumulation of misfolded protein aggregates is a hallmark event in many age-related protein misfolding disorders, including some of the most prevalent and insidious neurodegenerative diseases. Misfolded protein aggregates produce progressive cell damage, organ dysfunction, and clinical changes, which are common also in natural aging. Thus, we hypothesized that aging is associated to the widespread and progressive misfolding and aggregation of many proteins in various tissues. In this study, we analyzed whether proteins misfold, aggregate, and accumulate during normal aging in three different biological systems, namely senescent cells, *Caenorhabditis elegans*, and mouse tissues collected at different times from youth to old age. Our results show a significant accumulation of misfolded protein aggregates in aged samples as compared to young materials. Indeed, aged samples have between 1.3 and 2.5-fold (depending on the biological system) higher amount of insoluble proteins than young samples. These insoluble proteins exhibit the typical characteristics of disease-associated aggregates, including insolubility in detergents, protease resistance, and staining with amyloid-binding dye as well as accumulation in aggresomes. We identified the main proteins accumulating in the aging brain using proteomic studies. These results show that the aged brain contain large amounts of misfolded and likely non-functional species of many proteins, whose soluble versions participate in cellular pathways that play fundamental roles in preserving basic functions, such as protein quality control, synapsis, and metabolism. Our findings reveal a putative role for protein misfolding and aggregation in aging.

## Introduction

Aging can be defined as the widespread, gradual, and progressive decline of biological functions in an organism that leads to senescence and increases the risk of disease and death (López-Otín et al., 2013). The world’s population is aging. In the next 30 years, the group of people older than 60 years of age will increase sharply (Ortman et al., 2014). Moreover, aging is the strongest risk factor for many diseases. Therefore, there is an urgent need to understand the molecular and cellular mechanisms responsible for aging and to develop strategies to slow it.

Advanced age is a major risk factor for a clinically heterogeneous group of diseases, collectively called Protein Misfolding Disorders (PMDs; Kikis et al., 2010; Cuanalo-Contreras et al., 2013; Soto and Pritzkow, 2018). PMDs are caused by the misfolding, aggregation, and deposition of specific proteins in different tissues, leading to organ dysfunction and disease (Chiti and Dobson, 2006). Examples of disease-associated misfolded proteins are tau and amyloid beta in Alzheimer’s disease (AD), alpha-synuclein in Parkinson’s disease (PD), TDP43 and SOD1 in amyotrophic lateral sclerosis and amylin in type 2 diabetes (Mukherjee et al., 2015; Soto and Pritzkow, 2018). The process of protein misfolding and aggregation involves the formation of intermolecular beta-sheet polymers, which leads to insolubility and resistance to elimination by cellular clearance mechanisms (Sweeney et al., 2017). Proteins undergoing misfolding and aggregation deposit in various tissues, resulting in the loss of the normal biological function of the protein and/or the gain of a toxic activity (Winklhofer et al., 2008). The propensity to misfold is not exclusive of proteins associated to pathologies, as several non-disease related proteins bear sequences and three-dimensional arrangements that increase their susceptibility to misfold and aggregate (Goldschmidt et al., 2010; Soto, 2012). Since protein misfolding and aggregation can be induced by slight fluctuations in physiological conditions (Chiti and Dobson, 2009), we speculate that such alterations could trigger the changes that prompt these otherwise benign proteins to misfold and accumulate throughout life, contributing to the progressive dysfunction we called aging.

Advanced age imposes extra challenges on the proteome, as proteostasis undergoes a progressive decline during aging (Ben-Zvi et al., 2009; Taylor and Dillin, 2011). The Ubiquitin-Proteasome System (UPS) is one of the most important proteostasis network components (Amm, 2014). UPS is comprised of different enzymes that ubiquitinate proteins marking them for degradation by proteasomes. In both PMDs and aging, the UPS exhibits functional impairment, inciting a vicious cycle where misfolded proteins accumulate, thereby saturating and further impairing the proteostasis machinery (Saez and Vilchez, 2014; Vilchez et al., 2014). The latter promotes the sequestration of misfolded proteins into juxtanuclear inclusion bodies, called aggresome (Johnston et al., 1998). The presence of aggresome has been documented in several neurodegenerative PMDs (Bandopadhyay et al., 2005; Kristiansen et al., 2005; Olzmann et al., 2008; Santa-Maria et al., 2012).

Several studies have shown widespread accumulation of hundreds of insoluble proteins as a hallmark of aging in *Caenorhabditis elegans* (David et al., 2010; Reis-Rodrigues et al., 2012; Walther et al., 2015), *Saccharomyces cerevisiae* (Peters et al., 2012), *Mus musculus* heart (Ayyadevara et al., 2016), and *Nothobranchius furzeri* brain (Kelmer Sacramento et al., 2020). Yet, it remains unclear if these insoluble proteins have the hallmarks of misfolded protein aggregates associated to PMDs and whether they deposit over the course of a lifetime. We hypothesize that protein transition to insolubility during aging involves the misfolding, aggregation, and deposition of these proteins, analogous to what is observed in PMDs. In this study, we performed an extensive and systematic characterization of age-related aggregation (termed here *age-ggregation*) in different experimental models of natural aging, using a battery of established biochemical, histological, and proteomic techniques commonly employed in the PMDs field. Our results provide evidence of an extensive age-related accumulation of misfolded protein aggregates (*age-ggregates*) in *Caenorhabditis elegans*, Mouse Embryonic Fibroblasts (MEFs) and *Mus musculus* brain and heart, indicating that these insoluble aggregates may be associated to aging. In addition, we detected intracellular accumulation of misfolded proteins and aggresome formation in the aged brain. Our findings suggest that protein misfolding in the murine old brain has major physiological implications in synapsis, metabolism, proteostasis, and disease. Taken together, our observations reveal the magnitude of protein misfolding in aging and raise the question whether one important aspect of aging should be considered a similar process as in PMD.

## Materials and methods

### Sample collection and processing

#### Caenorhabditis elegans

We used the temperature-induced sterile *C. elegans* strain CF512: *rrf-3(b26) II; fem-1(hc17) IV,* obtained from the *Caenorhabditis Genetics Center* (CGC), University of Minnesota. To obtain synchronized cultures, we lysed adult hermaphrodites (0.25 M NaOH, 1% NaClO, 10 min) and collected their eggs. Eggs were washed in M9 solution (0.24 M Sodium Phosphate Dibasic Heptahydrate, 0.11 M Potassium Phosphate Monobasic, 40 mM sodium chloride, and 93 mM ammonium cloride) and larva 1 arrested overnight in S medium at 20°C. Worms were transferred and grow in NGM plates/*Escherichia coli* OP50 at 25°C during adult life until collection (days 1, 5, and 10). To remove bacterial debris that could interfere with our experiments, we washed the worms three times with distilled water (1,500 rpm for 1 min) and placed them for 10 min in a slow rocking shaker. Samples were homogenized (5,000 rpm for 15 s, 4x) in cold aqueous buffer (20 mM TRIS, 100 mM NaCl, 1X protease inhibitor cocktail from Roche) in presence of 0.5 mm glass beads using a *Precellys 24* homogenizer (Bertin instruments). Debris were removed (450 × *g* for 5 min at 4°C), and protein concentration was determined using Pierce MicroBCA Protein Assay Kit (ThermoFisher). Homogenates were stored at −80°C until use.

#### Mouse embryonic fibroblasts

We produced our in-house MEFs primary cultures, as previously described (Smith, 2006). Briefly, a pregnant B6C3F1 female was humanely sacrificed by exposure to CO_2_ at 14 days *post-coitum*, embryos were harvested and head, heart, and liver were removed. The remaining tissue was teased into fine pieces and digested overnight with 0.25% trypsin–EDTA at 4°C. On the next morning, trypsin was removed and a cell suspension was prepared by up and down pipetting. Debris were removed by sedimentation. The cell suspension was plated in 10-cm tissue culture dishes with MEFs culture medium (Dulbbeco’s DMEM from Sigma, 10% fetal bovine serum from Gibco, and 1X non-essential aminoacids from Gibco and 1X antimicotic-antibiotic from Gibco). MEFs were sequentially passaged once they reached 70–80% confluency. Cells were harvested at each passage and homogenized with a manual pestle in cold aqueous buffer. Debris were removed (450 × *g* for 5 min at 4°C), and protein concentration was determined using Pierce MicroBCA Protein Assay Kit (ThermoFisher). Homogenates were stored at −80°C until use.

To characterize MEFs senescence we measured population doubling (PD) levels, cellular size, and proliferation. To determine PD, we counted the total number of cells after each passage using a Neubauer chamber. PD was calculated as follows (Faraonio et al., 2012):


ΔPD=log(nh/ni)/log2

Where *nh* is the number of cells after passage, *ni* is the starting cell number.

To visualize and quantify cellular size, we stained 4% paraformaldehyde fixed permeabilized cells with ActinGreen 488 Ready Probes (ThermoFisher). Samples were mounted with Vectashield (Vector Labs) that contains DAPI to counterstain the nucleus.

To measure cell proliferation, we used the 5-Bromo-2′-deoxyuridine incorporation assay. Briefly, MEFs were treated for 24 h with a 10 μM solution of 5-Bromo-2′-deoxyuridine (BrdU, Sigma), followed by a 20 min fixation with Bouin’s solution (Sigma), and incubated overnight with G3G4 (anti-BrdU, Developmental studies hybridoma bank). We used Alexa Flour 594 (Life technologies) as secondary antibody. Samples were mounted with Vectashield (Vector Labs).

#### Mouse tissue samples

Brain, heart, liver, and intestine were harvested from PBS perfused male B6C3F1 mice of different ages (“young” 3 months old, “adult” 12 months old, and “old” 22 months old). Mice were housed at the University of Texas Health Science Center animal facility under standard conditions with a PicoLab Rodent 20 diet (Labdiet). Food and water were provided *ad libitum*. All animal experiments were approved by the University of Texas Health Science Center Animal Welfare Committee and were in accordance with NIH Guidelines. All homogenates were prepared in presence of cold aqueous buffer with 1X protease inhibitor cocktail from Roche; protein concentration was determined using Pierce MicroBCA Protein Assay Kit (ThermoFisher). Brains were homogenized using a Dounce homogenizer. Heart, liver, and intestine were homogenized (5,000 rpm for 20 s, 4x) in cold aqueous buffer with 2.8 mm stainless steel beads using a *Precellys 24* homogenizer (Bertin instruments). Debris were removed by low speed centrifugation (450 × *g* for 5 min at 4°C). Homogenates were stored at −80°C until use.

### Insoluble protein extraction

The extraction process was entirely performed at 4°C and in the presence of the protease inhibitor cocktail from Roche to avoid protein degradation. Samples were normalized by total protein concentration and treated for 1 h with 65 units/ml of Benzonase nuclease (Sigma) to remove nucleic acids and 1.35 units/mg of Lipase A (Sigma) to remove lipids. For insoluble protein extraction, we first centrifuged the sample at 20,000 × *g* for 1 h at 4°C in presence of aqueous buffer, followed by pellet resuspension and a second centrifugation (same speed as above) in aqueous buffer (wash). The pellet in aqueous solution was carefully resuspended in detergent buffer (20 mM TRIS–HCl, 100 mM NaCl, 0.1% SDS, 1% sodium deoxycholate, 1% Igepal, and 1X protease inhibitor cocktail from Roche) and centrifuged. The detergent insoluble pellet was washed two times with aqueous buffer to remove any residue of detergent that could interfere with our assays. Finally, the pellet was resuspended in aqueous buffer and mildly sonicated for 10 min at 100% Amplitude. We consider this suspension the insoluble fraction. Protein concentration was determined using Pierce MicroBCA Protein Assay Kit (ThermoFisher).

### Proteinase K resistance digestion assay

Samples were normalized by the concentration of insoluble protein. The insoluble fractions were digested with 0.3 μg of proteinase K (PK, Sigma) for each 20 μg of insoluble protein, at 37°C for 1 h. The digestion was stopped by the addition of 5 mM phenylmethylsulfonyl fluoride (PMSF, Sigma). We then precipitated the non-digested fraction by mixing the samples with four volumes of 100% methanol, followed by 1 h incubation at −80°C. The samples were centrifuged at 12,000 × *g* for 10 min and we determined the concentration of non-digested protein in the pellet using Pierce MicroBCA Protein Assay Kit (ThermoFisher). The percentage of undigested protein was determined using the following equation: Non digested protein after PK/total protein before PK × 100.

### Thioflavin T binding assay

Samples were normalized by the concentration of insoluble protein. Fluorescence readings of the insoluble fractions were performed in the presence of 5 μM thioflavin T (Sigma), using a Hitachi F7000 Fluorescence Spectrophotometer (excitation 435 nm, emission 485 nm). Fluorescence signal of the samples in absence of thioflavin T and a thioflavin T blank were used as background. For thioflavin T experiments in protein degraded samples, we subjected the insoluble fractions to a strong digestion treatment with 1 mg/ml proteinase K (PK, Sigma) for 1 h at 37°C, digestion was stopped with 5 mM phenylmethylsulfonyl fluoride (PMSF, Sigma). Digested products were mixed with 5 μM thioflavin T (Sigma), and fluorescence was determined as stated above.

### Histological analysis

Brain samples were fixed in formalin and paraffin embedded. 10-μm thick sections were obtained. After deparaffinization, we stained with thioflavin S, aggresome dye, and BTA-1. Briefly, thioflavin S (Sigma) staining was performed by incubation with a 0.1% thioflavin S solution for 10 min, followed by two washes in 80% ethanol. For BTA-1 staining, we incubated the samples with a 100 nm BTA-1 solution for 45 min, followed by a wash in distilled water. For aggresome staining, we used Proteostat Aggresome detection dye (Enzo Life Sciences) for 10 min, followed by a 20 min wash with 1% acetic acid. Samples were cover-slipped with Vectashield (Vector Labs) or Fluoro-Gel (EMS) mounting medium. Each group of samples was analyzed by immunofluorescence. Images were acquired with a Leica DMI 6000B microscope.

Confocal microscopy was performed at the Center for Advanced Microscopy, Department of Integrative Biology & Pharmacology University of Texas, Health Science Center. Brain histological sections were visualized using a Nikon A1R Confocal Laser Microscope System. The acquisition was made at standard high-resolution confocal mode. Image analysis and quantification was performed using Image J (RSB, Reserve Services Branch, NIH).

### Mass spectrometry

#### Sample preparation for mass spectrometry (MS)

Brain insoluble fractions were resuspended in 6 M guanidine hydrochloride, 0.1 M TRIS–HCl with 1X protease inhibitor cocktail from Roche, and sonicated for 4 min at 100% Amplitude. Samples were subjected to acetone precipitation; proteins were precipitated at −20°C overnight. After centrifugation (12,000 × *g* × 5 min), the pellets were resuspended in 10 μl of 150 mM Tris–HCl, pH 8.0, denatured and reduced with 20 μl of 9 M urea, 30 mM DTT in 150 mM Tris HCl, pH 8.0, at 37°C for 40 min, then alkylated with 40 mM iodacetamide in the dark for 30 min. The reaction mixture was diluted 10-fold using 50 mM Tris–HCl pH 8.0 prior to overnight digestion at 37°C with trypsin (1:20 enzyme to substrate ratio). Digestions were terminated with adding equal volume of 2% formic acid, and then desalted using a 1 ml reverse phase cartridges (Waters Oasis HLB) according to the vendor’s procedure: wash cartridge with 2 × 500 μl of 70% of acetonitrile in 0.1% formic acid, equilibrate with 2 × 500 μl of 0.1% formic acid, load total volume of digest, wash with 2 × 500 μl of 0.1% formic acid, and elute with 500 μl of 70% acetonitrile in 0.1% formic acid. Eluates were dried *via* vacuum centrifugation.

#### Liquid chromatography with tandem mass spectrometry analysis (LC/MS/MS)

An aliquot of the tryptic digest (in 2% acetonitrile/0.1% formic acid in water) was analyzed by LC/MS/MS on an Orbitrap Fusion™ Tribrid™ mass spectrometer (Thermo Scientific™) interfaced with a Dionex UltiMate 3000 Binary RSLCnano System. Peptides were separated onto a Acclaim™ PepMap ™ C_18_ column (75 μm ID × 15 cm, 2 μm) at a flow rate of 300 nl/min. Gradient conditions were: 3–22% B for 120 min; 22–35% B for 10 min; 35–90% B for 10 min; and 90% B held for 10 min (solvent A, 0.1% formic acid in water; solvent B, 0.1% formic acid in acetonitrile). The peptides were analyzed using data-dependent acquisition method, Orbitrap Fusion was operated with measurement of FTMS1 at resolutions 120,000 FWHM, scan range 350–1,500 m/z, AGC target 2E5, and maximum injection time of 50 ms; during a maximum 3 s cycle time, the ITMS2 spectra were collected at rapid scan rate mode, with CID NCE 35, 1.6 m/z isolation window, AGC target 1E4, maximum injection time of 35 ms, and dynamic exclusion was employed for 60 s.

#### Data processing and analysis

The raw data files were processed using Thermo Scientific™ Proteome Discoverer™ software version 1.4, spectra were searched against the IPI_mouse 3.87 database using the Mascot search engine. Search results were trimmed to a 1% FDR using Percolator. For the trypsin, up to two missed cleavages were allowed. MS tolerance was set 10 ppm; MS/MS tolerance 0.8 Da. Carbamidomethylation on cysteine residues was used as fixed modification; oxidation of methione as well as phosphorylation of serine, threonine and tyrosine was set as variable modifications.

### Functional annotation analysis

Functional annotation was performed using NIH-DAVID v6.8. We used *Mus musculus* genome as background list and the Kyoto Encyclopedia of Genes and Genomes biochemical pathways (KEGGs) for final analysis. Terms not relevant for brain physiology were removed. A value ≤0.05 of the modified Fisher Exact *p*-value (EASE Score) was considered significant.

### Statistical analysis

Statistics were performed with GraphPad Prism Software 5 (GraphPad Software, Inc.). Changes among groups were analyzed by *T*-test or one way ANOVA followed by Tukey *post-hoc* test. Value of *p* ≤ 0.05 was considered significant.

## Results

### Proteins misfold and accumulate during aging and cellular senescence

Misfolding and aggregation leads to protein insolubilization, resistance to elimination by cellular clearance and the ability of the aggregates to bind to certain amyloid-binding dyes (e.g., Thioflavin; Soto and Pritzkow, 2018). These properties have been frequently used to characterize protein aggregates in PMDs. We exploited these properties to study the occurrence of age-related protein misfolding at the level of the cell, organ, and full multicellular organism. For this purpose, we studied the presence and quantity of misfolded protein aggregates in senescent mouse embryonic fibroblasts (MEFs), aged mouse organs (brain, heart, liver, and intestine), and old *C. elegans*. The time to reach senescence and senescent properties in the MEF model were carefully evaluated by standard procedures (Supplementary Figure 1).

**Figure 1:**
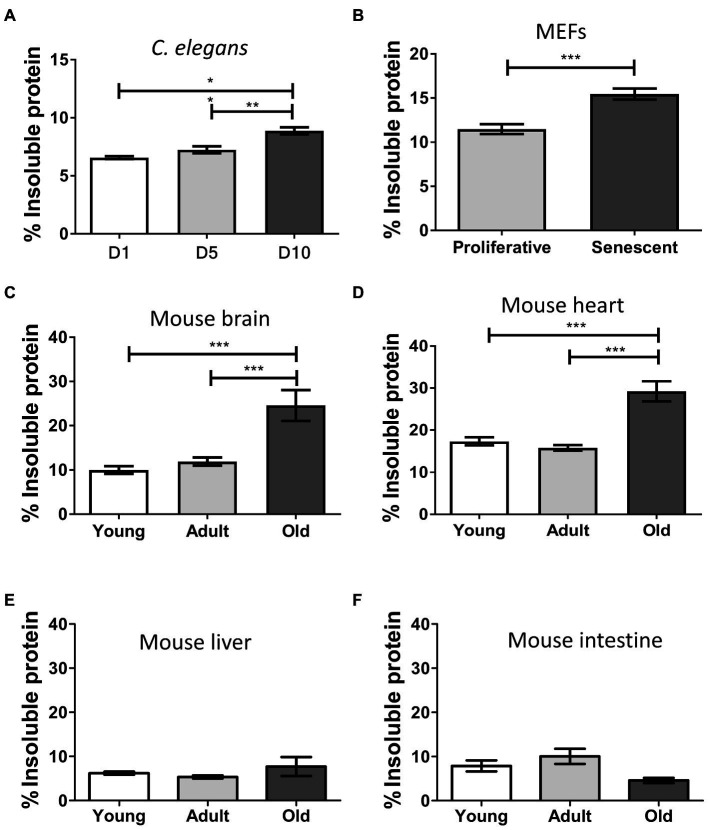
Insoluble protein levels increase during aging and senescence. Percentage of insoluble protein present in samples of: **(A)** *Caenorhabditis elegans*, day 1 (D1), day 5 (D5), and day 10 (D10) of adult life, *n* = 3; **(B)** MEFs, proliferative (population doubling or PD = 3), and senescent (PD = 8), *n* = 3; **(C)** Brain; **(D)** Heart; **(E)** Liver, and **(F)** Intestine of mouse, young (3 m.o., *n* = 8), adult (12 m.o., *n* = 8), and old (22 m.o., *n* = 4). One way ANOVA, Tukey *post-hoc* test. T-test (FEMs), ^ns^*p* > 0.05, ^*^p ≤ 0.05, ^**^p ≤ 0.01, and ^***^p ≤ 0.001. Average ± SEM.

Disease-associated misfolded aggregated proteins remain insoluble, even in the presence of strong detergents (Kushnirov et al., 2006; Yuan et al., 2006). To assess the levels of insoluble protein in our samples, we extracted and quantified the detergent-insoluble fractions from total homogenates and compared among different ages or proliferative stages (Figure 1). We observed a progressive and significant increase of insolubility during aging in *C. elegans* (*p* ≤ 0.01; Figure 1A), senescent MEFs (*p* ≤ 0.001; Figure 1B), and mouse brain and heart (*p* ≤ 0.001; Figures 1C,D). We detected low levels of insoluble proteins in young and adult samples, which remained nearly similar in both age groups. Comparing the means of young/proliferative with old/senescent samples, the most substantial fold-change occurred in mouse brain (2.5), followed by heart (1.7), MEFs (1.3), and worms (1.3). To our surprise, we did not detect statistical differences between young and old samples from liver or intestine (Figures 1E,F). These results suggest that the accumulation of misfolded protein aggregates during aging occurs only in some tissues.

**Figure 2:**
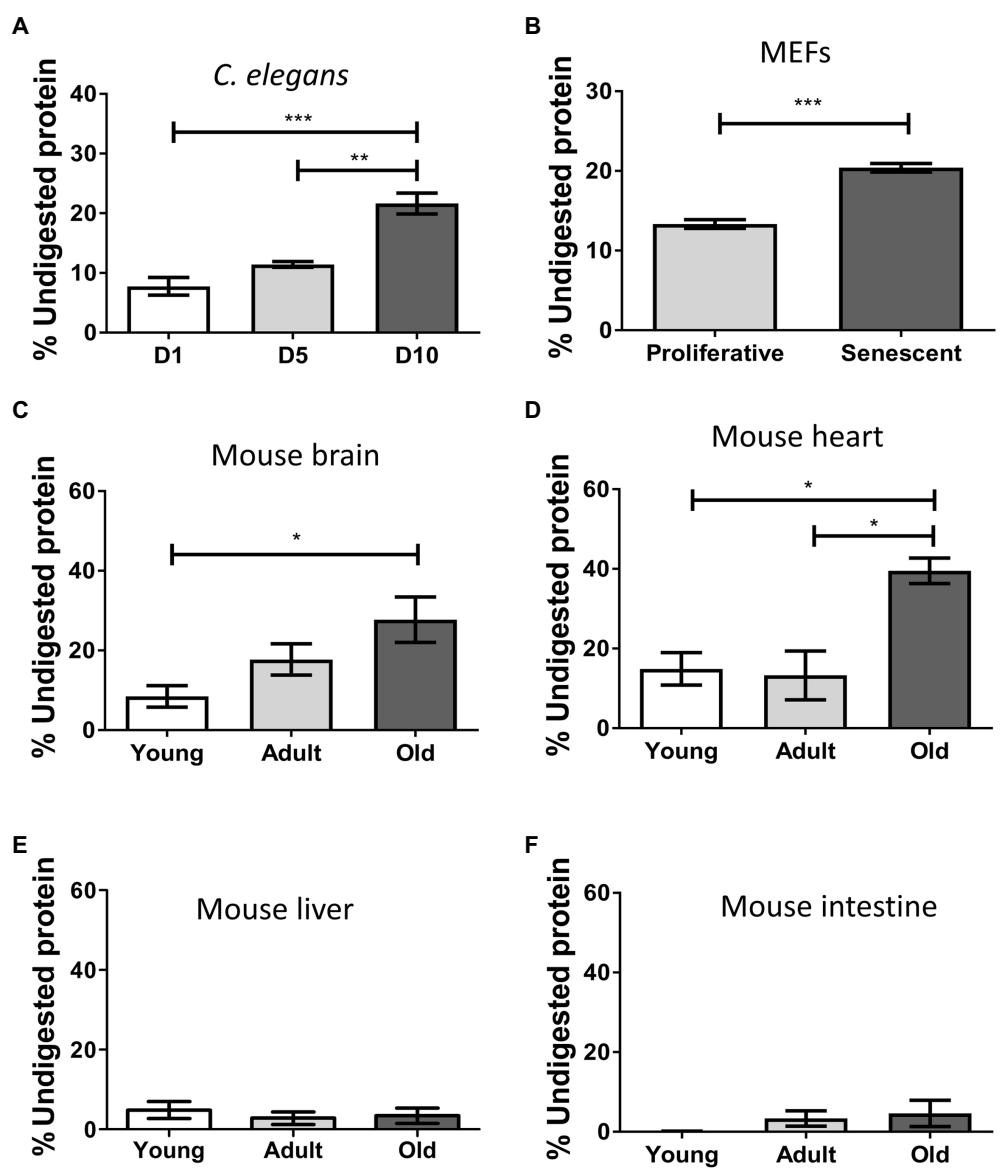
Insoluble proteins accumulating during aging and senescence are resistant to protease digestion. Percentage of protein resistant to PK digestion in the insoluble fraction of samples of: **(A)** *Caenorhabditis elegans*, day 1 (D1), day 5 (D5), and day 10 (D10) of adult life, *n* = 3; **(B)** MEFs, proliferative (PD = 3) and senescent (PD = 8), *n* = 3; **(C)** Brain; **(D)** Heart; **(E)** Liver; and **(F)** Intestine of mouse, young (3 m.o., *n* = 8), adult (12 m.o., *n* = 8), and old (22 m.o., *n* = 4). The percentage of protein remaining undigested was measure after precipitating proteins with methanol as described in the Methods. One way ANOVA, Tukey post-hoc test. T-test (FEMs), ^ns^*p* > 0.05, ^*^p ≤ 0.05, ^**^p ≤ 0.01, and ^***^p ≤ 0.001. Average ± SEM.

Misfolded protein aggregates are extremely resistant to thermal, chemical, or proteolytic degradation (Neumann et al., 2002; Stöhr et al., 2012; Saverioni et al., 2013). This property is critical for the high resistance of misfolded proteins to clearance, leading to accumulation and deposition. To test the ability of the insoluble proteome to resist proteolytic degradation, we performed controlled proteinase K (PK) digestion, followed by quantification of remaining proteins to determine the percentage of non-digested protein (Figure 2). We found the aged insoluble proteome of *C. elegans* (*p* ≤ 0.001; Figure 2A), senescent MEFs (*p* ≤ 0.001; Figure 2B), mouse brain (*p* ≤ 0.05; Figure 2C), and heart (*p* ≤ 0.05; Figure 2D), to be more resistant to digestion than their younger/proliferative counterparts. In terms of mean-fold increases, the highest contrast of young vs. old occurred in brain (3.3), followed by worms (2.8), heart (2.7), and senescent MEFs exhibited a modest increase (1.5). These results suggest that the aged insoluble proteome has an increased resistance to proteolytic digestion, consistent with the presence of aggregated structures. No significant changes on protease resistance in the liver or intestine proteome were observed (Figures 2E,F).

**Figure 3:**
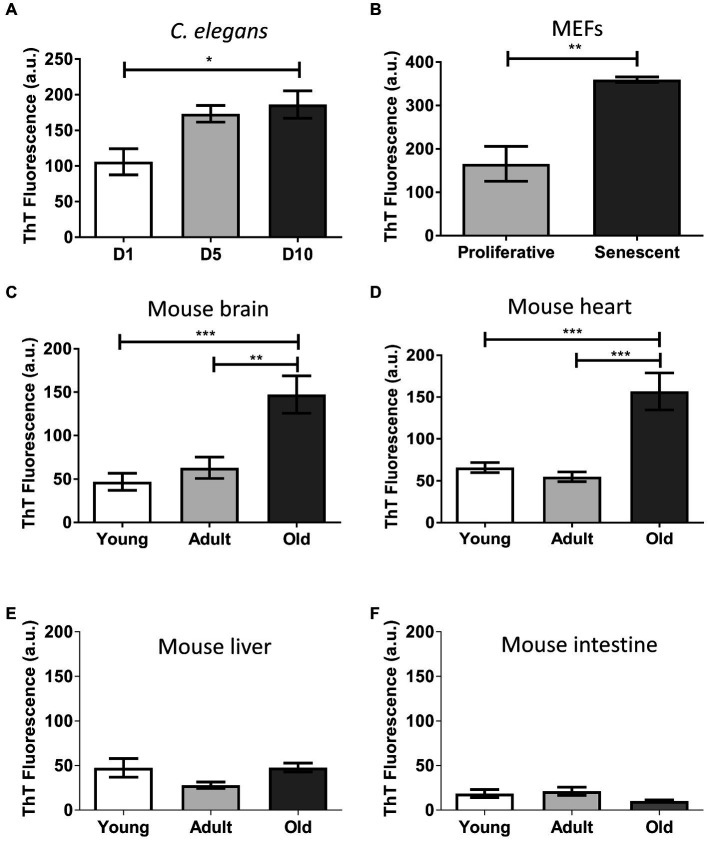
Insoluble proteins accumulated during aging and senescence bind to Thioflavin T. Thioflavin T fluorescence in arbitrary unit (a.u) of the insoluble fraction of: **(A)** *C. elegans*, day 1 (D1), day 5 (D5), and day 10 (D10) of adult life, *n* = 3; **(B)** MEFs, proliferative (PD = 3) and senescent (PD = 8), *n* = 3; **(C)** Brain; **(D)** Heart; **(E)** Liver; and **(F)** Intestine of mouse, young (3 m.o., *n* = 8), adult (12 m.o., *n* = 8), and old (22 m.o., *n* = 4). One way ANOVA, Tukey *post-hoc* test. T-test (FEMs), ^ns^*p* > 0.05, ^*^p ≤ 0.05, ^**^p ≤ 0.01, and ^***^p ≤ 0.001. Average ± SEM.

Thioflavin is a benzotiazolic molecule that exhibits an increase in fluorescence after binding to misfolded protein aggregates with high beta-sheet stacking (Xue et al., 2017). It has been extensively used for the *ex vivo* and *in vitro* detection of disease-associated misfolded aggregates (Saeed and Fine, 1967; LeVine, 1993; Rajamohamedsait and Sigurdsson, 2012; Xue et al., 2017). To gain insight on the supramolecular assembly present in the aged insoluble proteome, we performed *in vitro* thioflavin T (ThT) binding assays (Figure 3). To avoid nonspecific binding of the dye, we pre-treated the samples with DNase and lipase since DNA and lipids have been shown to bind ThT. The results show a significantly increased fluorescent signal in aged worms (*p* ≤ 0.05; Figure 3A), senescent MEFs (*p* ≤ 0.01; Figure 3B), old brain (*p* ≤ 0.001; Figure 3C), and old heart (*p* ≤ 0.001; Figure 3D). When we calculated the mean-fold increase of thioflavin signal of young vs. old, we found that aged brain insoluble proteome has the highest increase (3.1), followed by heart (2.4), MEFs (2.2), and worms (1.8). This result indicates the presence of an enriched fraction of misfolded proteins with intermolecular beta-sheet structure in the aged insoluble proteome. Again, we did not observe significant differences in the insoluble proteome of liver or intestine (Figures 3E,F). To exclude the possibility that ThT binding was associated to molecules other than proteins, we used a harsh digestion treatment (1 mg/ml protease for 1 h at 37°C) to degrade proteins in our samples. This treatment is often enough to degrade disease-associated amyloid proteins. We found little to no fluorescent signal upon treatment (Supplementary Figure 2). This result suggest that the ThT signal was indeed coming from misfolded protein aggregates. Thus, we conclude that during aging and senescence there are significantly high levels of misfolded protein aggregates.

### Extensive intracellular accumulation of misfolded proteins and aggresomes in aged brain

**Figure 4:**
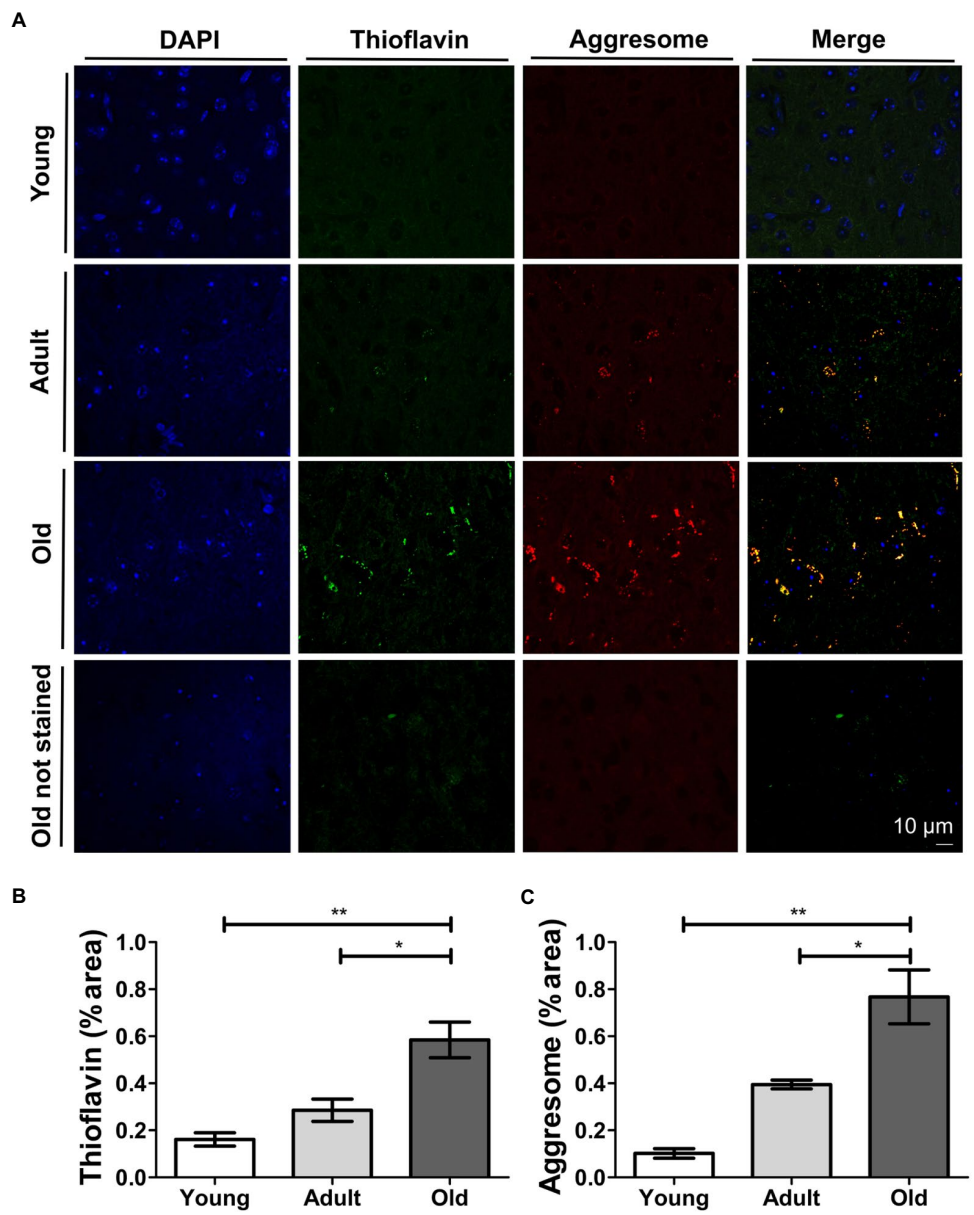
Thioflavin S and aggresome staining increase and co-localize in aged mouse brain. **(A)** Confocal microscopy of histological sections of mouse brain at different ages co-stained with Thioflavin S (green) and aggresome dye (red). Scale bar: 10 μm; Quantification of the percentage of area stained by Thioflavin S **(B)** and aggresome **(C)** dye in histological sections of mouse brain. Young (3 m.o.), adult (12 m.o.), and old (22 m.o.), *n* = 3 (an average of 20 slides per animal were analyzed). One way ANOVA, Tukey *post-hoc* test, ^ns^*p* > 0.05, ^*^p ≤ 0.05, ^**^p ≤ 0.01, and ^***^p ≤ 0.001. Average ± SEM.

Our biochemical results revealed remarkable increases in protein misfolding during aging in various systems, with the most clear effect seen in the mouse brain. Based on these results, we performed histological studies in mouse brain samples from the midbrain to gain a better understanding of the histopathology of non-disease-associated age-related accumulation of misfolded proteins. For this purpose, we evaluated the presence and location of misfolded proteins in brain slices of animals at different ages. We perfused and collected brains from young, adult, and naturally-aged *wild type* mice. We stained brain slices with thioflavin S (ThS). We did not detect extracellular ThS-positive deposits, as observed in AD brains (Bussiere et al., 2004). However, we observed a progressive intracellular punctuated-like ThS staining as age increased (Figures 4A,B). To confirm the ThS staining of old brain tissue indeed represents accumulation of misfolded protein aggregates, we used BTA-1, which is a blue fluorescent analog of ThS (Wu et al., 2008). As displayed in Supplementary Figure 3, this dye stained the same deposits as ThS. The particular juxtanuclear location of the staining caught our attention, as it indicates the possible sequestration of toxic misfolded proteins into aggresomes. Thus, we performed co-stainings with ThS and an aggresome detection dye (Figure 4A). We observed a good co-localization between thioflavin and aggresome signal (Figure 4A). We quantified the percentage of area stained by each of these procedures (Figures 4B,C) and found significant increase when we compared adult (*p* ≤ 0.01) and young (*p* ≤ 0.001) vs. the old group. We used confocal microscopy to avoid interference from the natural auto-fluorescence of the samples. Nevertheless, as shown in Figure 4A, old unstained tissue did not show significant auto-fluorescence. To further confirm that the fluorescent signal does not correspond to the background auto-fluorescence, we processed images for all samples with no staining (Supplementary Figure 4). Taken together, our results provide histological evidence of age-related intracellular accumulation of misfolded proteins sequestered into aggresomes in brain, in the absence of disease.

### Proteins involved in synapsis, metabolism, and proteostasis misfold in the aged brain

To unveil the identity of the proteins that misfold in the aged brain, we performed proteomic analysis of the insoluble fractions of the brain from young and aged mice. We analyzed only those proteins detected in all three individuals of each group. Using liquid chromatography–tandem mass spectrometry, we identified 1,101 proteins in the insoluble fraction of both young and old animals. To quantify protein abundance, we used peptide spectral match-based quantification and we calculated the old vs. young ratio for each protein. We found 168 proteins that consistently accumulate in the old insoluble proteome by 2-fold or more. We also identified 87 proteins exclusive to the aged proteome. Together, we call this set of 255 proteins, the aged-brain insoluble proteome. In addition, we found 27 insoluble proteins present only in young brains. Keratin is one of the proteins detected among the 255 proteins present in the aged insoluble proteome. However, keratin was not detected in the young insoluble proteome. This suggest that the presence of keratin is not due to experimental contamination, otherwise we would have detected this protein equally in old and young insoluble proteomes. The list of the proteins classes identified only in young brain insoluble proteome is displayed in Supplementary Table 1, and the list of all proteins in Supplementary Table 2.

To elucidate the processes affected by protein misfolding with advanced age, we looked within the aged-brain insoluble proteome for common pathways. We conducted a functional annotation analysis using NIH-DAVID v6.8 (Huang et al., 2009a,b) and explored the enriched KEGG pathways in the set (Kanehisa and Goto, 2000; Kanehisa et al., 2016, 2017). We found that proteins that misfold and accumulate in the aged brain play important biological roles in various key pathways, including dopaminergic synapsis, metabolism, proteostasis, fatty acid biosynthesis and degradation, endocytosis, and cytoskeleton (Table 1). Overall, our analysis shows that a specific set of proteins consistently misfold and accumulate with age in the brain, indicating that this is not a stochastic process, but rather a possible source of cellular dysfunction, resembling to what is observed in PMDs.

**Table 1:** Enriched KEGG pathways in the mouse aged brain insoluble proteome.

Biological pathway	*p* value	Fold Enrichment
Dopaminergic synapse	2.20E−04	4.8
Carbon metabolism	4.20E−04	5
Proteasome	6.20E−04	8.5
Fatty acid degradation	9.20E−04	7.8
Endocytosis	3.90E−03	2.8
Vasopressin-regulated water reabsorption	4.30E−03	7.4
Tight junction	5.80E−03	3.7
Biosynthesis of amino acids	7.10E−03	4.9
Fatty acid metabolism	7.90E−03	6.3
Metabolic pathways	1.10E−02	1.5
mRNA surveillance pathway	1.70E−02	4
Fatty acid biosynthesis	1.90E−02	13.7
Adrenergic signaling in cardiomyocytes	2.90E–02	3
Sphingolipid signaling pathway	4.40E−02	3.1
AMPK signaling pathway	4.70E−02	3
Regulation of actin cytoskeleton	4.90E−02	2.4

Analysis was carried out using the Database for Annotation, Visualization, and Integrated Discovery (DAVID) v6.8. A total of 121 out of 255 entries from the aged insoluble proteome were recognized by DAVID in the selected gene ontology functional annotation category. EASE score *p* value: modified Fisher exact *p* value (*p* ≤ 0.05).

## Discussion

The biological activity of cells and organisms depends on the proper function of many different proteins involved in key cellular signaling pathways. To remain biologically active, proteins need to preserve their native three-dimensional structure and solubility. Any alterations to these parameters challenge their ability to perform their normal biological function, with devastating consequences for the cell and the organism (Cuanalo-Contreras et al., 2013). Previously reported evidence showed a transition to insolubility of several proteins during aging in different models (David et al., 2010; Peters et al., 2012; Reis-Rodrigues et al., 2012; Ayyadevara et al., 2016; Kelmer Sacramento et al., 2020). Interestingly, many of these proteins are predicted to have high propensity to misfold and aggregate, similarly to PMDs (David et al., 2010). Based on these observations, we hypothesized that during aging several different proteins undergo progressive misfolding and aggregation to form structures similar to those found in age-related PMDs, causing widespread and chronic cellular dysfunction, which is the hallmark feature of aging (Cuanalo-Contreras et al., 2013).

In this study, we report the extensive and progressive accumulation of misfolded proteins during natural aging/senescence in different models, in the absence of disease. We coined the term *age-ggregates* to refer to this subset of proteins. Our findings demonstrate that age-ggregates exhibit the main characteristics of misfolded protein aggregates implicated in PMDs, including insolubility in detergents, protease-resistance, and staining with dyes specific for misfolded aggregates (i.e., thioflavin and BTA). Misfolded protein aggregates with these characteristics are thought to be implicated in some of today most prevalent diseases, including Alzheimer’s disease and related forms dementia, Parkinson’s disease, Amyotrophic Lateral Sclerosis, type 2 diabetes, and even cancer (Mukherjee et al., 2015; Soto and Pritzkow, 2018). The strongest risk factor for all these diseases is aging, supporting our concept that advanced age is associated with increased accumulation of misfolded protein aggregates. We found intracellular *age-ggregation* in the aged brain, where misfolded proteins are sequestered into aggresomes. Aggresomes have been studied in the context of neurodegenerative diseases, where they act as a general defense mechanism against high levels of accumulation of toxic misfolded proteins (Kopito, 2000). Our results indicate that the aged brain contains relatively large amounts of misfolded species, whose soluble versions participate in cellular pathways that play fundamental roles in preserving basic functions, such as protein quality control, synapsis, and metabolism. By comparison with PMD, it is likely that the aging-associated misfolded protein will be non-functional or acquire a toxic activity. Therefore, we speculate that age-related protein misfolding may play a key role in the decline of those processes. Alternatively, the formation of misfolded aggregates might be a consequence of a dysfunctional proteasomal and other degradation pathways. The reproducibility of our results using various different techniques, methodologies, and model systems (invertebrates, cellular models, and rodents) indicate that protein misfolding during aging is not a stochastic phenomenon, but rather that a specific subset of proteins are prone to misfold with age, in accordance with previous reports (David et al., 2010; Peters et al., 2012; Reis-Rodrigues et al., 2012). Several investigators have proposed that protein transition to insolubility in the context of aging is a conserved event (David et al., 2010; Peters et al., 2012); however, our results in aged liver and intestine question that paradigm. The fact that we did not observe *age-ggregation* increase in those organs, could indicate that their aging mechanism is intrinsically different, or that their proteome has less tendency to misfold or that they possess a more robust protein homeostasis system (Dasuri et al., 2009). It is interesting to note that liver and intestine are more mitotically active than the other models we tested (Miyaoka and Miyajima, 2013; Richardson et al., 2014), suggesting that age-related protein misfolding could be a hallmark of postmitotic/senescent age.

Our proteomic results show an over-representation of proteins implicated in proteostasis in the aged insoluble proteome of the brain, which is consistent with previous reports in other models (David et al., 2010; Peters et al., 2012; Reis-Rodrigues et al., 2012; Ayyadevara et al., 2016; Duncan et al., 2017). The proteostasis network play essential roles to clear damaged proteins. A general decline in proteostasis has been reported in aging and in several age-related disorders (Kikis et al., 2010; Morimoto and Cuervo, 2014). Confirming the role of protein homeostasis in aging, it has been shown that proteostasis activation leads to healthspan and lifespan extension (Harrison et al., 2009; Alavez et al., 2011; Ohtsuka et al., 2011; Vilchez et al., 2012). We found that proteins that misfold in the aged brain participate in several levels of the protein quality control system, from folding, to ubiquitin conjugation to degradation. Proteasome composition seems to be especially affected by brain age-ggregates, as we detected both regulatory and catalytic subunits of the proteasome in the insoluble proteome. Our observation of widespread *age-ggregation* of proteostasis elements is consistent with our detection of aggresomes in the aged brain and suggests that proteostasis is greatly impacted by misfolding at an advanced age.

Metabolism is another important function involved in brain health and function, and is impaired in the context of aging and neurodegeneration (Srivastava, 2019). Metabolic alterations have been linked to initiation and progression of cognitive decline, dementia, and neurovascular dysfunction (Camandola and Mattson, 2017). The brain is a highly energetic organ, it consumes 20% of the total energy expenditure of the body (Grimm and Eckert, 2017). This energy is primarily produced within the mitochondria (Grimm and Eckert, 2017). Mitochondrial fission is a very important process as it is involved in mitochondria distribution and mitophagy. Alterations in fission are linked to aging and neurodegeneration (Sebastián et al., 2016; Wai and Langer, 2016; Sebastián et al., 2017; Srivastava, 2017). Dynamin 1-like protein plays a key role in mitochondrial fission (van der Bliek et al., 2013), ensuring the survival of neurons (Kageyama et al., 2012). The fact that we detected this protein in the *age-ggregated* fraction, indicates that misfolding may affect mitochondrial dynamics and compromise neuronal viability. However, we did not study mitochondrial functionality or neuronal survival on these experiments. Nevertheless, our results are consistent with reports showing neuronal mitochondrial dysfunction during aging (Kowald and Kirkwood, 2000; Terman et al., 2010; Grimm and Eckert, 2017) and share similarities with previous studies pinpointing enrichment of insoluble mitochondrial proteins in old *C. elegans* (Reis-Rodrigues et al., 2012). We observed age-related misfolding of proteins involved in brain metabolism. We found several mitochondrial bioenergetic elements transitioning to insolubility, such as: ATP citrate lyase, acyl-CoA synthetase, aldehyde dehydrogenase 1 and 2, enoyl Coenzyme A hydratase, fatty acid synthase, glutathione S-transferase, hydroxyacyl-Coenzyme A dehydrogenase, among others. Several of them play roles in fatty acid metabolism, degradation, and biosynthesis, which are enriched in the insoluble proteome. Fatty acid metabolism influences many neurological functions, and plays anti-inflammatory and neuroprotective roles (Romano et al., 2017). Moreover, fatty acid metabolism is significantly dysregulated in neurodegenerative PMDs, such as AD (Snowden et al., 2017). The antioxidant defense of the mitochondria is affected during aging, as we found the oxidation resistance 1 protein and peroxiredoxin in the insoluble fraction. The antioxidant defense system of the mitochondria is extremely important for neuronal survival (Ferreira et al., 2019) and linked with aging (Nyström et al., 2012; Odnokoz et al., 2017). The oxidation resistance protein 1 overexpression protects against oxidative stress in models of neurodegenerative PMDs, and its deletion causes ataxia, neurodegeneration and lifespan decrease (Volkert et al., 2000; Oliver et al., 2011; Wu et al., 2016). Interestingly, we observed age-associated misfolding of dynamin 1-like (DNM1L), which is a protein involved in mitochondrial dynamics.

Not only proteostasis and metabolism are impaired during normal aging, but also there is a sustained decline on pre-and post-synaptic dopaminergic activity (Reeves et al., 2002). Dopamine plays crucial roles in the control of locomotor activity, learning, and memory (Puig et al., 2014). We propose that age-associated misfolding of proteins involved in dopaminergic synapsis could be responsible for this impairment, as we found kinesin 5B and 5C, calmodulin, and several catalytic and regulatory subunits of PP1 and PP2, in the brain insoluble aged proteome. Kinesin 5B and 5C transport vesicles to neuron terminals and its activity is dysregulated in neurodegenerative PMDs (Simunovic et al., 2009). Calmodulin binds to dopamine receptors to enhance their function (Liu et al., 2007) and PP1/PP2 regulate dopaminergic synapse (Nestler and Greengard, 1999). Interestingly, those proteins are also known mediators of ER stress (Szegezdi et al., 2006; Ozcan and Tabas, 2010), which is a defensive response against the accumulation of misfolded proteins and whose activity declines during aging and age-related diseases (Brown and Naidoo, 2012).

Unfolded protein response-mediated degradation of damaged proteins is an essential mechanism to maintain protein homeostasis and eliminate misfolded proteins in the brain (Ciechanover and Brundin, 2003). Interestingly, we found that UPS enzymes and proteasomal elements become insoluble with advanced age. We observed consistent age-related transition to insolubility of ubiquitin-like modifier activating enzyme 1 (which catalyzes ubiquitin conjugation of damaged proteins) and of several regulatory proteasomal subunits: Psmc5 (ATPase), Psmc4 (ATPase), Psmd2 (non-ATPase), as well as catalytic subunits: Psma3 (alpha type 3), Psma6 (alpha type 6), and Psma7 (alpha type 7). We detected misfolding of proteins that assist the folding of other proteins, such as chaperonin containing Tcp1.

Several proteins of our *age-ggregate* set overlap with the previously reported proteome of the insoluble fraction of aged mouse heart (Ayyadevara et al., 2016) as well as with proteins implicated in the formation of misfolded protein deposits in human neurodegenerative disorders (Table 2). These findings further corroborate the non-stochastic semi-conserved nature of the process and suggest that age-related protein misfolding plays significant roles in disease. We used the same selection parameters of our aged brain set to define the aged heart insoluble proteome (Ayyadevara et al., 2016), identifying a total of 385 proteins. We then compared both sets and were surprised by the overlap in biological pathways affected by age in both organs. We found a concurrence of proteins involved in dopaminergic synapse (serine/threonine-protein phosphatase 2A, Guanine nucleotide-binding protein G), fatty acid metabolism (enoyl-CoA hydratase, acyl coenzyme A thioester hydrolase), AMPK energy homeostasis (serine/threonine-protein phosphatase 2A, elongation factor 2), and proteostasis (proteasome subunit alpha type-6, 40S, and 60S ribosomal proteins). In addition, we found overlap in proteins involved in cell division (centromere protein V, cytoskeleton-associated protein 5, programmed cell death 6 interacting protein, and rho guanine nucleotide exchange factor 2). We also compared our set as well with the reported proteome of disease-associated human amyloid plaques (Liao et al., 2004), neurofibrillary tangles (NFT; Wang et al., 2005), and Lewy bodies (Xia et al., 2008). We identified the presence of tau protein in both diseased brain and our brain aging set. Microtubule associated protein tau misfolds and forms neurofibrillary tangles in AD brains and other tauopathies (Binder et al., 2005), where has been linked to toxicity, neurodegeneration, and cognitive decline (Gendron and Petrucelli, 2009). The diseased and aged sets of samples have a concomitant representation of proteins associated with mitochondrial function (Dynamin-1-like protein, Enoyl-CoA hydratase, and Peroxiredoxin-6) and proteostasis (Proteasome subunit alpha type-6, Ubiquitin carboxyl-terminal hydrolase isozyme L1); which illustrates the disease/aging link and highlights the influence of metabolism and protein quality control on these pathologies. In addition, we found the persistent presence of vimentin, an intermediate filament protein that forms the aggresome cage (Johnston et al., 1998).

**Table 2:** Overlap of mouse aged brain insoluble proteome with the insoluble proteome of mouse aged heart and human amyloid plaques, NFT, and Lewy bodies.

Protein	Overlap
40S ribosomal protein S15a	Mouse heart
60S ribosomal protein L23	Mouse heart
60S ribosomal protein L9	Mouse heart
Adenosylhomocysteinase	Mouse heart
Carbonic anhydrase 2	NFT
Carbonyl reductase [NADPH] 1	Lewy bodies/mouse heart
Centromere protein V	Mouse heart
Clathrin light chain A	Mouse heart
Complement C1q subcomponent subunit B	Mouse heart
Cytoskeleton-associated protein 5	Mouse heart
Cytosolic acyl coenzyme A thioester hydrolase	Mouse heart
Dynamin-1-like protein	Lewy bodies/mouse heart
Elongation factor 2	Mouse heart
Enoyl-CoA hydratase, mitochondrial	Mouse heart
Fascin	Mouse heart
Ferritin heavy chain	Mouse heart
Glial fibrillary acidic protein	Amyloid plaques
Glyoxalase domain-containing protein 4	Mouse heart
Guanine nucleotide-binding protein gamma-12	Mouse heart
Microtubule-associated protein tau	Lewy bodies/NFT
PC4 and SFRS1 interacting protein 1	Mouse heart
Peroxiredoxin-6	NFT/mouse heart
Phosphatidylethanolamine-binding protein 1	Mouse heart
Programmed cell death 6 interacting protein	Mouse heart
Proteasome subunit alpha type-6	Mouse heart
Rho GDP-dissociation inhibitor 1	Mouse heart
Rho guanine nucleotide exchange factor 2	Mouse heart
Serine/threonine-protein phosphatase 2A	Mouse heart
Protein phosphatase 2A catalytic subunit α-isoform	Mouse heart
T-complex protein 1 subunit beta	Mouse heart
Ubiquitin carboxyl-terminal hydrolase isozyme L1	NFT
Vimentin	NFT, amyloid plaques

Several proteins that misfold in the aged brain are represented in the aged heart (Ayyadevara et al., 2016) and disease associated set (Liao et al., 2004; Wang et al., 2005; Xia et al., 2008).

The 27 insoluble proteins exclusive to young brains are predominantly involved in development and morphogenesis (Supplementary Table 1), processes that become obsolete once a young animal is fully developed. This suggests that proteins that play a role early in life, become insoluble once out-to-date in young fully developed animals and are eventually eliminated later in life.

In summary, our findings show that protein misfolding, aggregation, and accumulation is not restricted to diseases, but is an integral component of “normal aging.” Our study provides new insights into non-stochastic protein misfolding during aging and imposes a new paradigm in the field. Based on our results, we propose that aging is caused by the progressive and widespread misfolding and aggregation of essential proteins and we regarded this as a pathological process, and, thus, we speculate that aging might be considered as a pathological process, similar to PMDs. Further investigation is required to understand the intrinsic factors governing *age-ggregation* and the differential susceptibility of tissues during the later stages of life and disease. We speculate that advanced age increases the susceptibility of a defined subset of proteins to misfold and that *age-ggregation* could be the pathological event behind aging in some, but not all, tissues. Our previous study showed that administration of inhibitors of protein misfolding and aggregation to *C.elegans* extended their lifespan and healthspan (Cuanalo-Contreras et al., 2017), supporting the link between protein misfolding, aggregation and aging. A deeper elucidation of the specific role of generalized protein misfolding in the context of advanced age will be required to determine whether *age-ggregation* is the consequence or the cause of aging. In this study, we identified a specific set of proteins that consistently misfold in the aged brain and that overlap with neurodegenerative diseases. This set of proteins could be used as targets toward the design of therapies to study and alleviate aging and age-related neurodegeneration. Finally, as has been noted, protein components of different theories of aging such as oxidative stress, metabolism, mitochondria, and protein homeostasis misfold during aging, suggesting that protein misfolding and aggregation could be the unifying mechanism that threads these theories all together.

## Data availability statement

The raw data supporting the conclusions of this article will be made available by the authors, without undue reservation.

## Ethics statement

The animal study was reviewed and approved by Institutional Animal Welfare Committee.

## Author contributions

KC-C carried out all the experiments, produced the final version of the figures, and prepared the draft of the manuscript. JS participated in the analysis of the proteomic results. AM and K-WP contributed with the experimental design and sample collection. EA contributed with the confocal microscopy image acquisition. CS is the principal investigator of the project and was responsible for coordinating research activity, analyzing the data, funding, and producing the final version of the article. All authors contributed to the article and approved the submitted version.

## Conflict of interest

The authors declare that the research was conducted in the absence of any commercial or financial relationships that could be construed as a potential conflict of interest.

## Publisher’s note

All claims expressed in this article are solely those of the authors and do not necessarily represent those of their affiliated organizations, or those of the publisher, the editors and the reviewers. Any product that may be evaluated in this article, or claim that may be made by its manufacturer, is not guaranteed or endorsed by the publisher.

## Supplementary material

The Supplementary material for this article can be found online at: https://www.frontiersin.org/articles/10.3389/fnagi.2022.1090109/full#supplementary-material

## References

[ref1] Alavez S. Vantipalli M. C. Zucker D. J. S. Klang I. M. Lithgow G. J. (2011). Amyloid-binding compounds maintain protein homeostasis during ageing and extend lifespan. Nature 472, 226–229. doi: 10.1038/nature09873, PMID: 21451522 PMC3610427

[ref2] Amm I. (2014). Protein quality control and elimination of protein waste: the role of the ubiquitin–proteasome system. Biochim. Biophys. Acta Mol. Cell Res. 1843, 182–196. doi: 10.1016/j.bbamcr.2013.06.031, PMID: 23850760

[ref3] Ayyadevara S. Mercanti F. Wang X. Mackintosh S. G. Tackett A. J. Prayaga S. V. S.. (2016). Age-and hypertension-associated protein aggregates in mouse heart have similar proteomic profiles. Hypertension 67, 1006–1013. doi: 10.1161/HYPERTENSIONAHA.115.06849, PMID: 26975704 PMC4833546

[ref4] Bandopadhyay R. Kingsbury A. E. Muqit M. M. Harvey K. Reid A. R. Kilford L.. (2005). Synphilin-1 and parkin show overlapping expression patterns in human brain and form aggresomes in response to proteasomal inhibition. Neurobiol. Dis. 20, 401–411. doi: 10.1016/j.nbd.2005.03.021, PMID: 15894486

[ref5] Ben-Zvi A. Miller E. A. Morimoto R. I. (2009). Collapse of proteostasis represents an early molecular event in Caenorhabditis elegans aging. Proc. Natl. Acad. Sci. 106, 14914–14919. doi: 10.1073/pnas.0902882106, PMID: 19706382 PMC2736453

[ref6] Binder L. I. Guillozet-Bongaarts A. L. Garcia-Sierra F. Berry R. W. (2005). Tau, tangles, and Alzheimer’s disease. Biochim. Biophys. Acta 1739, 216–223. doi: 10.1016/j.bbadis.2004.08.014 15615640

[ref7] Brown M. K. Naidoo N. (2012). The endoplasmic reticulum stress response in aging and age-related diseases. Front. Physiol. 3:263. doi: 10.3389/fphys.2012.00263 22934019 PMC3429039

[ref8] Bussiere T. Bard F. Barbour R. Grajeda H. Guido T. Khan K.. (2004). Morphological characterization of Thioflavin-S-positive amyloid plaques in transgenic Alzheimer mice and effect of passive Abeta immunotherapy on their clearance. Am. J. Pathol. 165, 987–995. doi: 10.1016/S0002-9440(10)63360-3, PMID: 15331422 PMC1618604

[ref9] Camandola S. Mattson M. P. (2017). Brain metabolism in health, aging, and neurodegeneration. EMBO J. 36, 1474–1492. doi: 10.15252/embj.201695810, PMID: 28438892 PMC5452017

[ref10] Chiti F. Dobson C. M. (2006). Protein Misfolding, functional amyloid, and human disease. Annu. Rev. Biochem. 75, 333–366. doi: 10.1146/annurev.biochem.75.101304.123901 16756495

[ref11] Chiti F. Dobson C. M. (2009). Amyloid formation by globular proteins under native conditions. Nat. Chem. Biol. 5, 15–22. doi: 10.1038/nchembio.131 19088715

[ref12] Ciechanover A. Brundin P. (2003). The ubiquitin proteasome system in neurodegenerative diseases: sometimes the chicken, sometimes the egg. Neuron 40, 427–446. doi: 10.1016/S0896-6273(03)00606-8 14556719

[ref13] Cuanalo-Contreras K. Mukherjee A. Soto C. (2013). Role of protein misfolding and proteostasis deficiency in protein misfolding diseases and aging. Int. J. Cell Biol. 2013:638083. doi: 10.1155/2013/638083 24348562 PMC3855986

[ref14] Cuanalo-Contreras K. Park K. W. Mukherjee A. Millán-Pérez Peña L. Soto C. (2017). Delaying aging in Caenorhabditis elegans with protein aggregation inhibitors. Biochem. Biophys. Res. Commun. 482, 62–67. doi: 10.1016/j.bbrc.2016.10.143, PMID: 27810360

[ref15] Dasuri K. Nguyen A. Zhang L. Fernandez-Kim O. S. Bruce-Keller A. J. Blalock B. A.. (2009). Comparison of rat liver and brain proteasomes for oxidative stress-induced inactivation: influence of ageing and dietary restriction. Free Radic. Res. 43, 28–36. doi: 10.1080/10715760802534812, PMID: 19048434 PMC2735019

[ref16] David D. C. Ollikainen N. Trinidad J. C. Cary M. P. Burlingame A. L. Kenyon C. (2010). Widespread protein aggregation as an inherent part of aging in C. elegans. PLoS Biol. 8, 47–48. doi: 10.1371/journal.pbio.1000450 PMC2919420 20711477

[ref17] Duncan F. E. Jasti S. Paulson A. Kelsh J. M. Fegley B. Gerton J. L. (2017). Age-associated dysregulation of protein metabolism in the mammalian oocyte. Aging Cell 16, 1381–1393. doi: 10.1111/acel.12676, PMID: 28994181 PMC5676066

[ref18] Faraonio R. Salerno P. Passaro F. Sedia C. Iaccio A. Bellelli R.. (2012). A set of miRNAs participates in the cellular senescence program in human diploid fibroblasts. Cell Death Differ. 19, 713–721. doi: 10.1038/cdd.2011.143, PMID: 22052189 PMC3307984

[ref19] Ferreira J. C. B. Mori M. A. Gross E. R. (2019). Mitochondrial bioenergetics and quality control mechanisms in health and disease. Oxidative Med. Cell. Longev. 2019:5406751. doi: 10.1155/2019/5406751 PMC6360560 30805083

[ref20] Gendron T. F. Petrucelli L. (2009). The role of tau in neurodegeneration. Mol. Neurodegener. 4:13. doi: 10.1186/1750-1326-4-13, PMID: 19284597 PMC2663562

[ref21] Goldschmidt L. Teng P. K. Riek R. Eisenberg D. (2010). Identifying the amylome, proteins capable of forming amyloid-like fibrils. Proc. Natl. Acad. Sci. 107, 3487–3492. doi: 10.1073/pnas.0915166107, PMID: 20133726 PMC2840437

[ref22] Grimm A. Eckert A. (2017). Brain aging and neurodegeneration: from a mitochondrial point of view. J. Neurochem. 143, 418–431. doi: 10.1111/jnc.14037, PMID: 28397282 PMC5724505

[ref23] Harrison D. E. Strong R. Sharp Z. D. Nelson J. F. Astle C. M. Flurkey K.. (2009). Rapamycin fed late in life extends lifespan in genetically heterogeneous mice. Nature 460, 392–395. doi: 10.1038/nature08221, PMID: 19587680 PMC2786175

[ref24] Huang D. W. Sherman B. T. Lempicki R. A. (2009a). Systematic and integrative analysis of large gene lists using DAVID bioinformatics resources. Nat. Protoc. 4, 44–57. doi: 10.1038/nprot.2008.211, PMID: 19131956

[ref25] Huang D. W. Sherman B. T. Lempicki R. A. (2009b). Bioinformatics enrichment tools: paths toward the comprehensive functional analysis of large gene lists. Nucleic Acids Res. 37, 1–13. doi: 10.1093/nar/gkn923, PMID: 19033363 PMC2615629

[ref26] Johnston J. A. Ward C. L. Kopito R. R. (1998). Aggresomes: a cellular response to misfolded proteins. J. Cell Biol. 143, 1883–1898. doi: 10.1083/jcb.143.7.1883, PMID: 9864362 PMC2175217

[ref27] Kageyama Y. Zhang Z. Roda R. Fukaya M. Wakabayashi J. Wakabayashi N.. (2012). Mitochondrial division ensures the survival of postmitotic neurons by suppressing oxidative damage. J. Cell Biol. 197, 535–551. doi: 10.1083/jcb.201110034, PMID: 22564413 PMC3352955

[ref28] Kanehisa M. Furumichi M. Tanabe M. Sato Y. Morishima K. (2017). KEGG: new perspectives on genomes, pathways, diseases and drugs. Nucleic Acids Res. 45, D353–D361. doi: 10.1093/nar/gkw1092, PMID: 27899662 PMC5210567

[ref29] Kanehisa M. Goto S. (2000). KEGG: Kyoto encyclopedia of genes and genomes. Nucleic Acids Res. 28, 27–30. doi: 10.1093/nar/28.1.27, PMID: 10592173 PMC102409

[ref30] Kanehisa M. Sato Y. Kawashima M. Furumichi M. Tanabe M. (2016). KEGG as a reference resource for gene and protein annotation. Nucleic Acids Res. 44, D457–D462. doi: 10.1093/nar/gkv1070, PMID: 26476454 PMC4702792

[ref31] Kelmer Sacramento E. Kirkpatrick J. M. Mazzetto M. Baumgart M. Bartolome A. Di Sanzo S.. (2020). Reduced proteasome activity in the aging brain results in ribosome stoichiometry loss and aggregation. Mol. Syst. Biol. 16:e9596. doi: 10.15252/msb.20209596 32558274 PMC7301280

[ref32] Kikis E. A. Gidalevitz T. Morimoto R. I. (2010). Protein homeostasis in models of aging and age-related conformational disease. Adv. Exp. Med. Biol. 694, 138–159. doi: 10.1007/978-1-4419-7002-2_11, PMID: 20886762 PMC3402352

[ref33] Kopito R. R. (2000). Aggresomes, inclusion bodies and protein aggregation. Trends Cell Biol. 10, 524–530. doi: 10.1016/S0962-8924(00)01852-3 11121744

[ref34] Kowald A. Kirkwood T. B. L. (2000). Accumulation of defective mitochondria through delayed degradation of damaged organelles and its possible role in the ageing of post-mitotic and dividing cells. J. Theor. Biol. 202, 145–160. doi: 10.1006/jtbi.1999.1046, PMID: 10640434

[ref35] Kristiansen M. Messenger M. J. Klöhn P.-C. Brandner S. Wadsworth J. D. F. Collinge J.. (2005). Disease-related prion protein forms aggresomes in neuronal cells leading to caspase activation and apoptosis. J. Biol. Chem. 280, 38851–38861. doi: 10.1074/jbc.M506600200, PMID: 16157591

[ref36] Kushnirov V. V. Alexandrov I. M. Mitkevich O. V. Shkundina I. S. Ter-Avanesyan M. D. (2006). Purification and analysis of prion and amyloid aggregates. Methods 39, 50–55. doi: 10.1016/j.ymeth.2006.04.007 16774835

[ref37] LeVine H. (1993). Thioflavine T interaction with synthetic Alzheimer's disease beta-amyloid peptides: detection of amyloid aggregation in solution. Protein Sci. 2, 404–410. doi: 10.1002/pro.5560020312, PMID: 8453378 PMC2142377

[ref38] Liao L. Cheng D. Wang J. Duong D. M. Losik T. G. Gearing M.. (2004). Proteomic characterization of postmortem amyloid plaques isolated by laser capture microdissection. J. Biol. Chem. 279, 37061–37068. doi: 10.1074/jbc.M403672200, PMID: 15220353

[ref40] Liu Y. Buck D. C. Macey T. A. Lan H. Neve K. A. (2007). Evidence that calmodulin binding to the dopamine D2 receptor enhances receptor signaling. J. Recept. Signal Transduct. Res. 27, 47–65. doi: 10.1080/10799890601094152, PMID: 17365509

[ref01] López-Otín C. Blasco M. A. Partridge L. Serrano M. Kroemer G. (2013). The hallmarks of aging. Cell, 153, 1194–1217. doi: 10.1016/j.cell.2013.05.039 23746838 PMC3836174

[ref41] Miyaoka Y. Miyajima A. (2013). To divide or not to divide: revisiting liver regeneration. Cell Div 8:8. doi: 10.1186/1747-1028-8-8, PMID: 23786799 PMC3695844

[ref42] Morimoto R. I. Cuervo A. M. (2014). Proteostasis and the aging proteome in health and disease. AJ. Gerontol. Ser. A. Biol. Sci. Med. Sci. 69, S33–S38. doi: 10.1093/gerona/glu049 PMC4022129 24833584

[ref43] Mukherjee A. Morales-Scheihing D. Butler P. C. Soto C. (2015). Type 2 diabetes as a protein misfolding disease. Trends Mol. Med. 21, 439–449. doi: 10.1016/j.molmed.2015.04.005, PMID: 25998900 PMC4492843

[ref44] Nestler E. J. Greengard P. (1999). “Protein serine-threonine phosphatases” in Basic Neurochemistry: Molecular, Cellular and Medical Aspects. 6th Edn. eds. Siegel G. J. Agranoff B. W. Albers R. W. Fisher S. K. Uhler M. D. (Lippincott-Raven)

[ref45] Neumann M. Kahle P. J. Giasson B. I. Ozmen L. Borroni E. Spooren W.. (2002). Misfolded proteinase K-resistant hyperphosphorylated alpha-synuclein in aged transgenic mice with locomotor deterioration and in human alpha-synucleinopathies. J. Clin. Invest. 110, 1429–1439. doi: 10.1172/JCI200215777, PMID: 12438441 PMC151810

[ref46] Nyström T. Yang J. Molin M. (2012). Peroxiredoxins, gerontogenes linking aging to genome instability and cancer. Genes Dev. 26, 2001–2008. doi: 10.1101/gad.200006.112, PMID: 22987634 PMC3444726

[ref47] Odnokoz O. Nakatsuka K. Klichko V. I. Nguyen J. Solis L. C. Ostling K.. (2017). Mitochondrial peroxiredoxins are essential in regulating the relationship between drosophila immunity and aging. Biochim. Biophys. Acta 1863, 68–80. doi: 10.1016/j.bbadis.2016.10.017 PMC5154953 27770625

[ref48] Ohtsuka H. Azuma K. Murakami H. Aiba H. (2011). hsf1 + extends chronological lifespan through Ecl1 family genes in fission yeast. Mol. Gen. Genomics. 285, 67–77. doi: 10.1007/s00438-010-0588-6, PMID: 21072667

[ref49] Oliver P. L. Finelli M. J. Edwards B. Bitoun E. Butts D. L. Becker E. B. E.. (2011). Oxr1 is essential for protection against oxidative stress-induced neurodegeneration. PLoS Genet. 7:e1002338. doi: 10.1371/journal.pgen.1002338, PMID: 22028674 PMC3197693

[ref50] Olzmann J. A. Li L. Chin L. S. (2008). Aggresome formation and neurodegenerative diseases: therapeutic implications. Curr. Med. Chem. 15, 47–60. doi: 10.2174/092986708783330692, PMID: 18220762 PMC4403008

[ref51] Ortman J.M. Velkoff V.A. Hogan H. (2014). An aging nation: The older population in the United States: Population estimates and projections. Current Population Report.

[ref52] Ozcan L. Tabas I. (2010). Pivotal role of calcium/calmodulin-dependent protein kinase II in ER stress-induced apoptosis. Cell Cycle 9, 223–224. doi: 10.4161/cc.9.2.10596, PMID: 20023415 PMC2846633

[ref54] Peters T. W. Rardin M. J. Czerwieniec G. Evani U. S. Reis-Rodrigues P. Lithgow G. J.. (2012). Tor1 regulates protein solubility in Saccharomyces cerevisiae. Mol. Biol. Cell 23, 4679–4688. doi: 10.1091/mbc.e12-08-0620, PMID: 23097491 PMC3521677

[ref56] Puig M. V. Rose J. Schmidt R. Freund N. (2014). Dopamine modulation of learning and memory in the prefrontal cortex: insights from studies in primates, rodents, and birds. Front. Neural Circuits 8:93. doi: 10.3389/fncir.2014.00093 25140130 PMC4122189

[ref57] Rajamohamedsait H. B. Sigurdsson E. M. (2012). Histological staining of amyloid and pre-amyloid peptides and proteins in mouse tissue. Methods Mol. Biol. 849, 411–424. doi: 10.1007/978-1-61779-551-0_28, PMID: 22528106 PMC3859432

[ref58] Reeves S. Bench C. Howard R. (2002). Ageing and the nigrostriatal dopaminergic system. Int. J. Geriatr. Psychiatry 17, 359–370. doi: 10.1002/gps.606 11994891

[ref59] Reis-Rodrigues P. Czerwieniec G. Peters T. W. Evani U. S. Alavez S. Gaman E. A.. (2012). Proteomic analysis of age-dependent changes in protein solubility identifies genes that modulate lifespan. Aging Cell 11, 120–127. doi: 10.1111/j.1474-9726.2011.00765.x, PMID: 22103665 PMC3437485

[ref60] Richardson R. B. Allan D. S. Le Y. (2014). Greater organ involution in highly proliferative tissues associated with the early onset and acceleration of ageing in humans. Exp. Gerontol. 55, 80–91. doi: 10.1016/j.exger.2014.03.015, PMID: 24685641

[ref61] Romano A. Koczwara J. B. Gallelli C. A. Vergara D. Micioni Di Bonaventura M. V. Gaetani S.. (2017). Fats for thoughts: An update on brain fatty acid metabolism. Int. J. Biochem. Cell Biol. 84, 40–45. doi: 10.1016/j.biocel.2016.12.015, PMID: 28065757

[ref62] Saeed S. M. Fine G. (1967). Thioflavin-T for amyloid detection. Am. J. Clin. Pathol. 47, 588–593. doi: 10.1093/ajcp/47.5.588 4164576

[ref63] Saez I. Vilchez D. (2014). The mechanistic links between proteasome activity, aging and age-related diseases. Curr. Genomics 15, 38–51. doi: 10.2174/138920291501140306113344, PMID: 24653662 PMC3958958

[ref64] Santa-Maria I. Varghese M. Ksiezak-Reding H. Dzhun A. Wang J. Pasinetti G. M. (2012). Paired helical filaments from Alzheimer disease brain induce intracellular accumulation of tau protein in aggresomes. J. Biol. Chem. 287, 20522–20533. doi: 10.1074/jbc.M111.323279, PMID: 22496370 PMC3370237

[ref65] Saverioni D. Notari S. Capellari S. Poggiolini I. Giese A. Kretzschmar H. A.. (2013). Analyses of protease resistance and aggregation state of abnormal prion protein across the spectrum of human prions. J. Biol. Chem. 288, 27972–27985. doi: 10.1074/jbc.M113.477547, PMID: 23897825 PMC3784711

[ref66] Sebastián D. Palacín M. Zorzano A. (2017). Mitochondrial dynamics: coupling mitochondrial fitness with healthy aging. Trends Mol. Med. 23, 201–215. doi: 10.1016/j.molmed.2017.01.003, PMID: 28188102

[ref67] Sebastián D. Sorianello E. Segalés J. Irazoki A. Ruiz-Bonilla V. Sala D.. (2016). Mfn2 deficiency links age-related sarcopenia and impaired autophagy to activation of an adaptive mitophagy pathway. EMBO J. 35, 1677–1693. doi: 10.15252/embj.201593084, PMID: 27334614 PMC4969577

[ref68] Simunovic F. Yi M. Wang Y. Macey L. Brown L. T. Krichevsky A. M.. (2009). Gene expression profiling of substantia nigra dopamine neurons: further insights into Parkinson’s disease pathology. Brain 132, 1795–1809. doi: 10.1093/brain/awn323, PMID: 19052140 PMC2724914

[ref69] Smith C. L. (2006). Mammalian Cell Culture. Curr. Protoc. Mol. Biol. 73, 28.0.1–28.0.2. doi: 10.1002/0471142727.mb2800s73

[ref70] Snowden S. G. Ebshiana A. A. Hye A. An Y. Pletnikova O. O’Brien R.. (2017). Association between fatty acid metabolism in the brain and Alzheimer disease neuropathology and cognitive performance: a nontargeted metabolomic study. PLoS Med. 14:e1002266. doi: 10.1371/journal.pmed.1002266, PMID: 28323825 PMC5360226

[ref71] Soto C. (2012). Protein misfolding and disease; protein refolding and therapy. FEBS Lett. 498, 204–207. doi: 10.1016/S0014-5793(01)02486-3 11412858

[ref72] Soto C. Pritzkow S. (2018). Unfolding the role of protein misfolding in neurodegenerative diseases. Nat. Rev. Neurosci. 4, 49–60. doi: 10.1038/nrn1007 12511861

[ref73] Srivastava S. (2017). The mitochondrial basis of aging and age-related disorders. Gene 8:398. doi: 10.3390/genes8120398, PMID: 29257072 PMC5748716

[ref74] Srivastava S. (2019). Emerging insights into the metabolic alterations in aging using metabolomics. Meta 9:301. doi: 10.3390/metabo9120301, PMID: 31847272 PMC6950098

[ref75] Stöhr J. Watts J. C. Mensinger Z. L. Oehler A. Grillo S. K. DeArmond S. J.. (2012). Purified and synthetic Alzheimer’s amyloid beta (Aβ) prions. Proc. Natl. Acad. Sci. U. S. A. 109, 11025–11030. doi: 10.1073/pnas.1206555109, PMID: 22711819 PMC3390876

[ref76] Sweeney P. Park H. Baumann M. Dunlop J. Frydman J. Kopito R.. (2017). Protein misfolding in neurodegenerative diseases: implications and strategies. Transl. Neurodegener. 6:6. doi: 10.1186/s40035-017-0077-5, PMID: 28293421 PMC5348787

[ref77] Szegezdi E. Logue S. E. Gorman A. M. Samali A. (2006). Mediators of endoplasmic reticulum stress-induced apoptosis. EMBO Rep. 7, 880–885. doi: 10.1038/sj.embor.7400779, PMID: 16953201 PMC1559676

[ref78] Taylor R. C. Dillin A. (2011). Aging as an event of proteostasis collapse. Cold Spring Harb. Perspect. Biol. 3:a004440. doi: 10.1101/cshperspect.a004440 21441594 PMC3101847

[ref79] Terman A. Kurz T. Navratil M. Arriaga E. A. Brunk U. T. (2010). Mitochondrial turnover and aging of long-lived Postmitotic cells: the mitochondrial–lysosomal axis theory of aging. Antioxid. Redox Signal. 12, 503–535. doi: 10.1089/ars.2009.2598, PMID: 19650712 PMC2861545

[ref80] van der Bliek A. M. Shen Q. Kawajiri S. (2013). Mechanisms of mitochondrial fission and fusion. Cold Spring Harb. Perspect. Biol. 5:a011072. doi: 10.1101/cshperspect.a011072 23732471 PMC3660830

[ref81] Vilchez D. Morantte I. Liu Z. Douglas P. M. Merkwirth C. Rodrigues A. P. C.. (2012). RPN-6 determines C. elegans longevity under proteotoxic stress conditions. Nature 489, 263–268. doi: 10.1038/nature11315, PMID: 22922647

[ref82] Vilchez D. Saez I. Dillin A. (2014). The role of protein clearance mechanisms in organismal ageing and age-related diseases. Nat. Commun. 5:5659. doi: 10.1038/ncomms6659, PMID: 25482515

[ref83] Volkert M. R. Elliott N. A. Housman D. E. (2000). Functional genomics reveals a family of eukaryotic oxidation protection genes. Proc. Natl. Acad. Sci. U. S. A. 97, 14530–14535. doi: 10.1073/pnas.260495897, PMID: 11114193 PMC18953

[ref84] Wai T. Langer T. (2016). Mitochondrial dynamics and metabolic regulation. Trends Endocrinol. Metab. 27, 105–117. doi: 10.1016/j.tem.2015.12.001 26754340

[ref85] Walther D. M. Kasturi P. Zheng M. Pinkert S. Vecchi G. Ciryam P.. (2015). Widespread proteome remodeling and aggregation in Aging C. elegans. Cells 161, 919–932. doi: 10.1016/j.cell.2015.03.032, PMID: 25957690 PMC4643853

[ref86] Wang Q. Woltjer R. L. Cimino P. J. Pan C. Montine K. S. Zhang J.. (2005). Proteomic analysis of neurofibrillary tangles in Alzheimer disease identifies GAPDH as a detergent-insoluble paired helical filament tau binding protein. FASEB J. 19, 869–871. doi: 10.1096/fj.04-3210fje, PMID: 15746184

[ref87] Winklhofer K. F. Tatzelt J. Haass C. (2008). The two faces of protein misfolding: gain-and loss-of-function in neurodegenerative diseases. EMBO J. 27, 336–349. doi: 10.1038/sj.emboj.7601930, PMID: 18216876 PMC2234348

[ref88] Wu Y. Davies K. E. Oliver P. L. (2016). The antioxidant protein Oxr1 influences aspects of mitochondrial morphology. Free Radic. Biol. Med. 95, 255–267. doi: 10.1016/j.freeradbiomed.2016.03.029, PMID: 27036366 PMC4891067

[ref89] Wu C. Wang Z. Lei H. Duan Y. Bowers M. T. Shea J. E. (2008). The binding of thioflavin T and its neutral analog BTA-1 to protofibrils of the Alzheimer's disease Abeta(16-22) peptide probed by molecular dynamics simulations. J. Mol. Biol. 384, 718–729. doi: 10.1016/j.jmb.2008.09.062, PMID: 18851978 PMC2712570

[ref90] Xia Q. Liao L. Cheng D. Duong D. M. Gearing M. Lah J. J.. (2008). Proteomic identification of novel proteins associated with Lewy bodies. Front. Biosci. 13, 3850–3856. doi: 10.2741/2973, PMID: 18508479 PMC2663966

[ref91] Xue C. Lin T. Y. Chang D. Guo Z. (2017). Thioflavin T as an amyloid dye: fibril quantification, optimal concentration and effect on aggregation. R. Soc. Open Sci. 4:160696. doi: 10.1098/rsos.160696, PMID: 28280572 PMC5319338

[ref92] Yuan J. Xiao X. McGeehan J. Dong Z. Cali I. Fujioka H.. (2006). Insoluble aggregates and protease-resistant conformers of prion protein in uninfected human brains. J. Biol. Chem. 281, 34848–34858. doi: 10.1074/jbc.M602238200, PMID: 16987816

