# Supplementary material for: Extensive accumulation of misfolded protein aggregates during natural aging and senescence
Source: Front Aging Neurosci. 2023 Jan 26;14:1090109. doi: 10.3389/fnagi.2022.1090109 (PMC9909609; doi:10.3389/fnagi.2022.1090109)
Supplement: Supplementary file 1 [file Data_Sheet_1.pdf]

Supplementary Materials

Supplementary Table 1. Enriched biological processes in the young mouse brain insoluble proteome. Analysis was carried out using the Database for Annotation, Visualization and Integrated Discovery (DAVID) v6.8. EASE score p value: modified Fisher exact p value ($p \leq 0.05$).

Term	P-Value	Fold Enrichment
**Neuron projection development**	**9.20E-03**	**19.5**
**Calcium-mediated signaling**	**1.80E-02**	**106.4**
**Protein targeting to mitochondrion**	**2.30E-02**	**82.2**
**Mitochondrion morphogenesis**	**3.30E-02**	**56.5**
**Nervous system development**	**5.90E-02**	**7.2**

Supplementary Table 2; List of all insoluble proteins in the aging proteome

Accession	Description
Q11011	Puromycin-sensitive aminopeptidase
Q3TZ32	Putative uncharacterized protein
Q8R0Y6	Aldehyde dehydrogenase family 1 member L1
Q8C0M9	L-asparaginase
E9Q6Q4	Uncharacterized protein
Q9R0P5	Destrin
O08709	Peroxiredoxin-6
Q3TED3	Putative uncharacterized protein
Q3TP62	Pleckstrin homology domain containing, family B (Evectins) member 1, isoform CRA_b
Q9R0P9	Ubiquitin carboxyl-terminal hydrolase isozyme L1
Q8BL66	Early endosome antigen 1
A3KME3	Kbtbd11 protein
A2AGT5	Cytoskeleton-associated protein 5
P16054	Protein kinase C epsilon type
Q05DT0	Add3 protein (Fragment)
O55023	Inositol monophosphatase 1
Q9ES97	Reticulon-3 OS=Mus musculus GN=Rtn3 PE=1 SV=2 - [RTN3_MOUSE]
Q9WV34	MAGUK p55 subfamily member 2
Q3TEU8	Putative uncharacterized protein
Q3TPI3	Putative uncharacterized protein (Fragment)
Q5BL10	Kinesin family member 5B
A2A7X3	Ubiquitin-conjugating enzyme
Q6ZQ38	Cullin-associated NEDD8-dissociated protein 1
Q6P5F9	Exportin-1
Q8K1M6	Dynamin-1-like protein
P10637	Microtubule-associated protein tau
Q9CS42	Ribose-phosphate pyrophosphokinase 2
Q91V12	Cytosolic acyl coenzyme A thioester hydrolase
Q80TL0	Protein phosphatase 1E
Q3TE06	Putative uncharacterized protein (Fragment)
B1AQW3	Microtubule-associated protein
P28738	Kinesin heavy chain isoform 5C
Q8R3V5	Endophilin-B2
Q4KMM3	Oxidation resistance protein 1
E9PUD2	Uncharacterized protein
Q5EBJ4	Ermin
Q99P72	Reticulon-4
Q76MZ3	Serine/threonine-protein phosphatase 2A 65 kDa regulatory subunit A alpha isoform
Q9Z0E0	Neurochondrin
Q60829	Protein phosphatase 1 regulatory subunit 1B

Q8C437	PEX5-related protein
P51410	60S ribosomal protein L9
E9QM49	Uncharacterized protein
Q8BGT8	Phytanoyl-CoA hydroxylase-interacting protein-like
Q547J4	Microtubule-associated protein
Q9R0Y5	Adenylate kinase isoenzyme 1
E9QP92	Uncharacterized protein
Q9CZC8	Secernin-1
Q8K0S0	Phytanoyl-CoA hydroxylase-interacting protein
Q61553	Fascin
O70161	Phosphatidylinositol-4-phosphate 5-kinase type-1 gamma
Q3TJ97	Putative uncharacterized protein
Q3UZJ4	Putative uncharacterized protein
Q52KE3	Gnal1 protein (Fragment)
Q6NZL0	Uncharacterized protein C6orf174 homolog
E9Q2A6	Uncharacterized protein
Q5SQB0	Nucleophosmin 1
Q9CYT6	Adenylyl cyclase-associated protein
Q00PI9	Heterogeneous nuclear ribonucleoprotein U-like protein 2
Q3TT18	Putative uncharacterized protein
P14106	Complement C1q subcomponent subunit B
Q7TQD2	Tubulin polymerization-promoting protein
P61922	4-aminobutyrate aminotransferase, mitochondrial
Q99PU5	Long-chain-fatty-acid--CoA ligase ACSBG1
P58252	Elongation factor 2
E9Q2W9	Uncharacterized protein
Q3UF82	Putative uncharacterized protein
Q02053	Ubiquitin-like modifier-activating enzyme 1
Q3U8N1	Putative uncharacterized protein
Q60668	Heterogeneous nuclear ribonucleoprotein D0
Q99LC5	Electron transfer flavoprotein subunit alpha, mitochondrial
Q8K0T0	Reticulon-1
Q3TFD9	Putative uncharacterized protein
Q3TI27	Ribose-phosphate pyrophosphokinase
P70296	Phosphatidylethanolamine-binding protein 1
E9PZV5	Uncharacterized protein
E9QAC7	Uncharacterized protein
P03995	Glial fibrillary acidic protein
A2AN08	E3 ubiquitin-protein ligase UBR4
Q9D6R2	Isocitrate dehydrogenase [NAD] subunit alpha, mitochondrial
Q9D394	Protein RUFY3
Q80SW1	Putative adenosylhomocysteinase 2
E9PZ90	Uncharacterized protein

P09528	Ferritin heavy chain
Q9Z140	Copine-6
P47738	Aldehyde dehydrogenase, mitochondrial
O35098	Dihydropyrimidinase-related protein 4
E9Q7H5	Uncharacterized protein
P19096	Fatty acid synthase
Q9QYG0	Protein NDRG2
P62196	26S protease regulatory subunit 8
Q8C522	Endonuclease domain-containing 1 protein
Q8C5R8	Ribose-phosphate pyrophosphokinase
P61161	Actin-related protein 2
Q99JY0	Trifunctional enzyme subunit beta, mitochondrial
P57780	Alpha-actinin-4
Q5FW75	Actinin alpha 2
Q7M6W1	RTN1-C
Q9Z0H8	CAP-Gly domain-containing linker protein 2
E9PUE7	Uncharacterized protein
E9QK48	Uncharacterized protein
P80318	T-complex protein 1 subunit gamma
Q8K232	Adducin 1 (Alpha)
Q99PT1	Rho GDP-dissociation inhibitor 1
P63005	Platelet-activating factor acetylhydrolase IB subunit alpha
Q3TGW0	Putative uncharacterized protein
Q8BH66	Atlastin-1
Q3UYN2	Putative uncharacterized protein
P00920	Carbonic anhydrase 2
Q3TI61	Putative uncharacterized protein
Q9JME5	AP-3 complex subunit beta-2
Q7TT10	Adaptor-related protein complex 2, sigma 1 subunit
Q9QZE5	Coatomer subunit gamma
Q3U0C4	Putative uncharacterized protein
E9Q105	Uncharacterized protein
E9Q3M3	Uncharacterized protein
Q9QUM9	Proteasome subunit alpha type-6
Q3TED2	Programmed cell death 6 interacting protein
P62204	Calmodulin
P27546	Microtubule-associated protein 4
P62748	Hippocalcin-like protein 1
A2ALS5	Rap1 GTPase-activating protein
Q61699	Heat shock protein 105 kDa
Q9QYB8	Beta-adducin
E9QM56	Uncharacterized protein
Q5SUT0	Ewing sarcoma breakpoint region 1

D3Z0M7	Dynein cytoplasmic 1 intermediate chain 1, isoform CRA_a
Q8BH80	Vesicle-associated membrane protein, associated protein B and C
Q9R0Q6	Actin-related protein 2/3 complex subunit 1A
P80313	T-complex protein 1 subunit eta
Q3TFE8	Putative uncharacterized protein
A1BN54	Alpha actinin 1a
O35954	Membrane-associated phosphatidylinositol transfer protein 1
Q3UCX7	Putative uncharacterized protein
P62830	60S ribosomal protein L23
P35278	Ras-related protein Rab-5C
Q9EQF6	Dihydropyrimidinase-related protein 5
P35700	Peroxiredoxin-1
Q7TPR4	Alpha-actinin-1
P62137	Serine/threonine-protein phosphatase PP1-alpha catalytic subunit
P28663	Beta-soluble NSF attachment protein
Q9EPN1	Neurobeachin
Q3TTF6	Putative uncharacterized protein
P70333	Heterogeneous nuclear ribonucleoprotein H2
P49615	Cyclin-dependent kinase 5
Q8R2D4	MCG140223]
Q3UNH4	G protein-regulated inducer of neurite outgrowth 1
E9Q7S0	Uncharacterized protein
Q3U781	MCG21131, isoform CRA_a
Q9D7X3	Dual specificity protein phosphatase 3
A2AD25	MCG49690
O70435	Proteasome subunit alpha type-3
Q9D6C8	Putative uncharacterized protein
Q3THL2	Putative uncharacterized protein
E9QKZ6	Uncharacterized protein
Q9CYH2	UPF0765 protein C10orf58 homolog
Q5SXY1	Cytospin-B
A2AKX3	Probable helicase senataxin
Q9JJI8	60S ribosomal protein L38
Q9CXS4	Centromere protein V
E9QNW9	Uncharacterized protein
E9QMF3	Uncharacterized protein
D4N6R6	Beta-globin
A2AMH3	Solute carrier family 44, member 1
Q9CX86	Putative uncharacterized protein
P63330	Serine/threonine-protein phosphatase 2A catalytic subunit alpha isoform
Q9DBJ1	Phosphoglycerate mutase 1
Q3TKM5	Isocitrate dehydrogenase 3 (NAD+), gamma, isoform CRA_c
Q62348	Translin

P40240	CD9 antigen
E9QA32	Uncharacterized protein
Q8CGA0	Protein phosphatase 1F
D3Z7I9	Oxysterol-binding protein
Q8C550	Putative uncharacterized protein
Q3UNZ8	Quinone oxidoreductase-like protein 2
Q9R1R9	UBE-1a
Q8VCW8	Acyl-CoA synthetase family member 2, mitochondrial
Q32MV2	Keratin 82
B2RWW1	Microtubule-associated protein 1S
B1AXF2	Solute carrier family 24 (Sodium/potassium/calcium exchanger), member 2
O54984	ATPase Asna1
E9QNK4	Uncharacterized protein
Q80XK8	Nbas protein (Fragment)
B1B0C7	Perlecan (Heparan sulfate proteoglycan 2)
A2BI13	PC4 and SFRS1 interacting protein 1 (Fragment)
Q9CU60	Putative uncharacterized protein (Fragment)
Q99LD4	COP9 signalosome complex subunit 1
Q8CE29	Ubiquitin protein ligase E3A
Q9D1P4	Cysteine and histidine-rich domain-containing protein 1
Q9CVY8	Putative uncharacterized protein
B2KFD4	Aspartoacylase (Aminoacylase) 2
E9Q197	Uncharacterized protein
O88543	COP9 signalosome complex subunit 3
E9PX61	Uncharacterized protein
Q8CCS7	Calbindin 2, isoform CRA_a
Q3TA40	Putative uncharacterized protein
E9Q460	Uncharacterized protein
Q99K51	Plastin-3
Q9DAS9	Guanine nucleotide-binding protein G(I)/G(S)/G(O) subunit gamma-12
B8JJ84	Syntaxin binding protein 6 (Amisyn) (Fragment)
Q5M8N4	Epimerase family protein SDR39U1
E9Q5Z3	Uncharacterized protein
Q99J09	Methylosome protein 50
Q05BD4	Prpsap2 protein
Q9CRY7	Glycerophosphodiester phosphodiesterase domain-containing protein 1
Q9CRE9	Putative uncharacterized protein (Fragment)
O35449	Proline-rich transmembrane protein 1
Q61081	Hsp90 co-chaperone Cdc37
Q3TDM2	Putative uncharacterized protein
E9PVZ5	Uncharacterized protein
P97390	Vacuolar protein sorting-associated protein 45
P46664	Adenylosuccinate synthetase isozyme 2

E9QPD4	Uncharacterized protein
B2KFH8	Parkinson disease (Autosomal recessive, early onset) 7
O09172	Glutamate--cysteine ligase regulatory subunit
Q3THK7	GMP synthase [glutamine-hydrolyzing]
Q99KC8	von Willebrand factor A domain-containing protein 5A
Q3V1M8	Putative uncharacterized protein
D3YWK1	Uncharacterized protein
Q99KJ8	Dynactin subunit 2
Q8BWG8	Beta-arrestin-1
Q60676	Serine/threonine-protein phosphatase 5
Q91ZP9	N-terminal EF-hand calcium-binding protein 2
Q3TEA8	Heterochromatin protein 1-binding protein 3
Q3TDN2	FAS-associated factor 2
P12658	Calbindin
Q9D358	Low molecular weight phosphotyrosine protein phosphatase
Q8K2U0	Map2k4 protein
Q8BN05	Putative uncharacterized protein (Fragment)
Q3TW74	Putative uncharacterized protein
Q6DI95	Transportin 3
P19157	Glutathione S-transferase P 1
Q8BH95	Enoyl-CoA hydratase, mitochondrial
E9PV04	Uncharacterized protein
D3YYM6	Uncharacterized protein
Q3U6U7	Putative uncharacterized protein
Q80Y17	Lethal(2) giant larvae protein homolog 1
O55091	Protein IMPACT
Q3UTI7	Putative uncharacterized protein
Q8CAA7	Glucose 1,6-bisphosphate synthase
Q91VB8	Alpha globin 1]
B9EHZ5	Membrane protein, palmitoylated 6 (MAGUK p55 subfamily member 6)
P84086	Complexin-2
Q8R4V2	Dual specificity protein phosphatase 15
Q8C7C4	Putative uncharacterized protein (Fragment)
Q9DBP5	UMP-CMP kinase
Q3UM23	Putative uncharacterized protein
Q99K69	RAS protein activator like 1 (GAP1 like)
Q8K1J7	Acyl-CoA synthetase long-chain family member 3
P48758	Carbonyl reductase [NADPH] 1
A3KGQ6	Actin-related protein 2/3 complex subunit 5
Q5UE59	Kinesin light chain-1
P15331	Peripherin
P35235	Tyrosine-protein phosphatase non-receptor type 11
P28474	Alcohol dehydrogenase class-3

Supplementary Figure 1. MEFs senescence characterization. A) MEFs population doubling (PD) curve**,** arrows indicate the samples used in this study; **B)** Phalloidin staining and cellular size quantification in proliferative and senescent MEFs. DAPI (blue), phalloidin (green), n= 30 cells; **C)** 5-Bromo-2′-deoxyuridine (BrdU) incorporation in proliferative and senescent MEFs. DAPI (blue), BrdU (red), n=100 cells. T test, ns $P > 0.05$, $*P \leq 0.05$, $**P \leq 0.01$, $***P \leq 0.001$. Average ± SEM. Scale bar 200 µm.

Supplementary Figure 2. Thioflavin T binding was eliminated by harsh proteolytic degradation. Samples of insoluble proteins were treated with a high concentration of PK (1mg/ml) for 1h at 37 ºC. Thioflavin T units of fluorescence was measured as described in methods of: **A)** *C. elegans*, day 1 (D1), day 5 (D5) and day 10 (D10) of adult life, n=3; **B)** MEFs, proliferative (PD=3) and senescent (PD=8), n=3; **C)** Brain; **D)** Heart; **E)** Liver and **F)** Intestine of mouse, young (3 m.o., n=8), adult (12 m.o., n=8) and old (22 m.o., n=4). One way ANOVA, Tukey post-hoc test. Average ± SEM.

Supplementary Figure 3. A) Confocal microscopy of histological sections of old mouse brain (22 m.o.), co-stained with Thioflavin S (green), aggresome dye (red) and thioflavine analog BTA-1 (blue); **B)** Co-localization of Thioflavin S, aggresome dye and BTA-1 in old (22 m.o.) mouse brain. Scale bar: 10 µm.

Supplementary Figure 4. Confocal microscopy of non-stained histological sections of young (3 m.o.), adult (12 m.o.) and old (22 m.o.) mouse brain. Scale bar: 10 µm

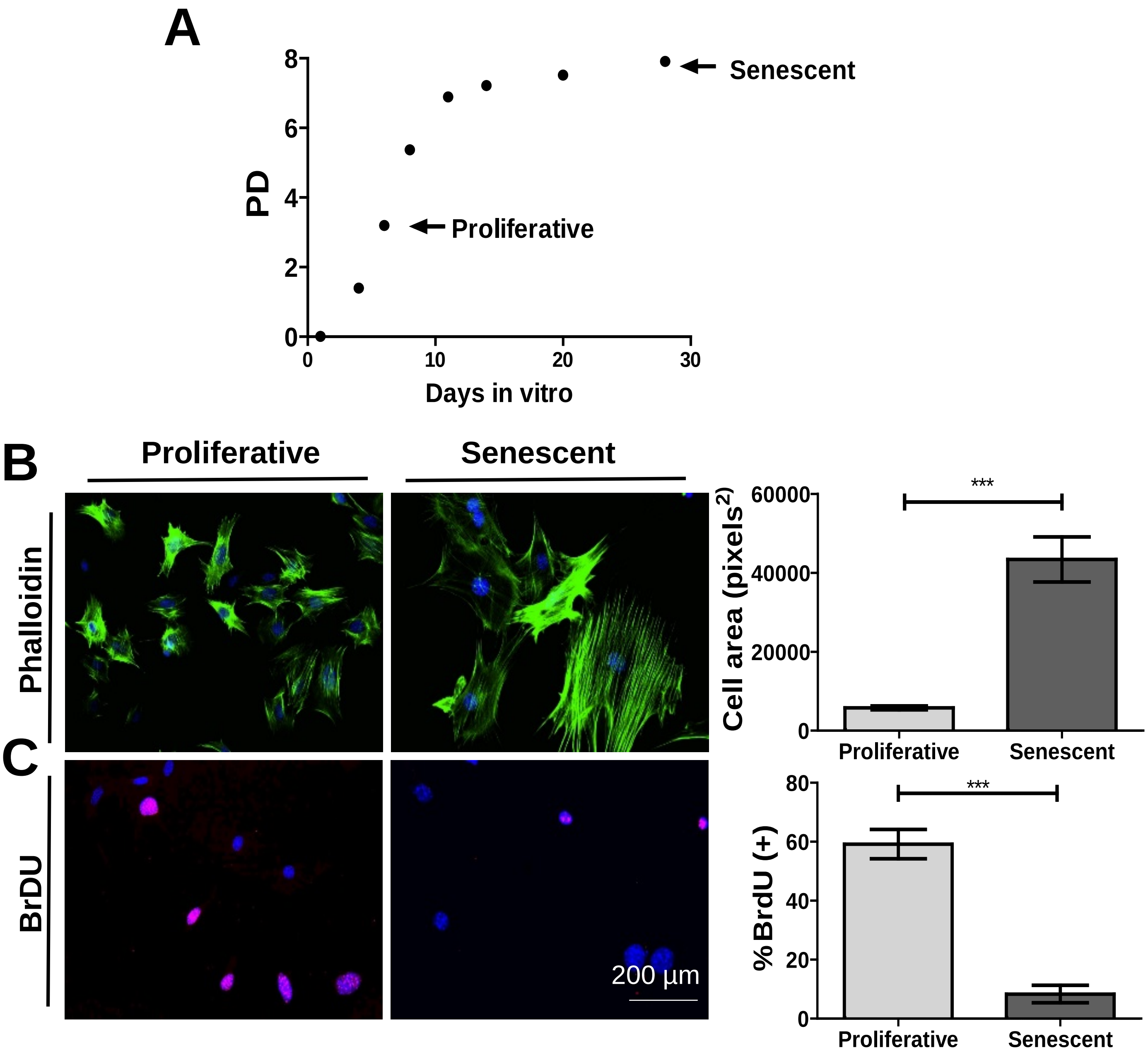

Supplementary Figure 1

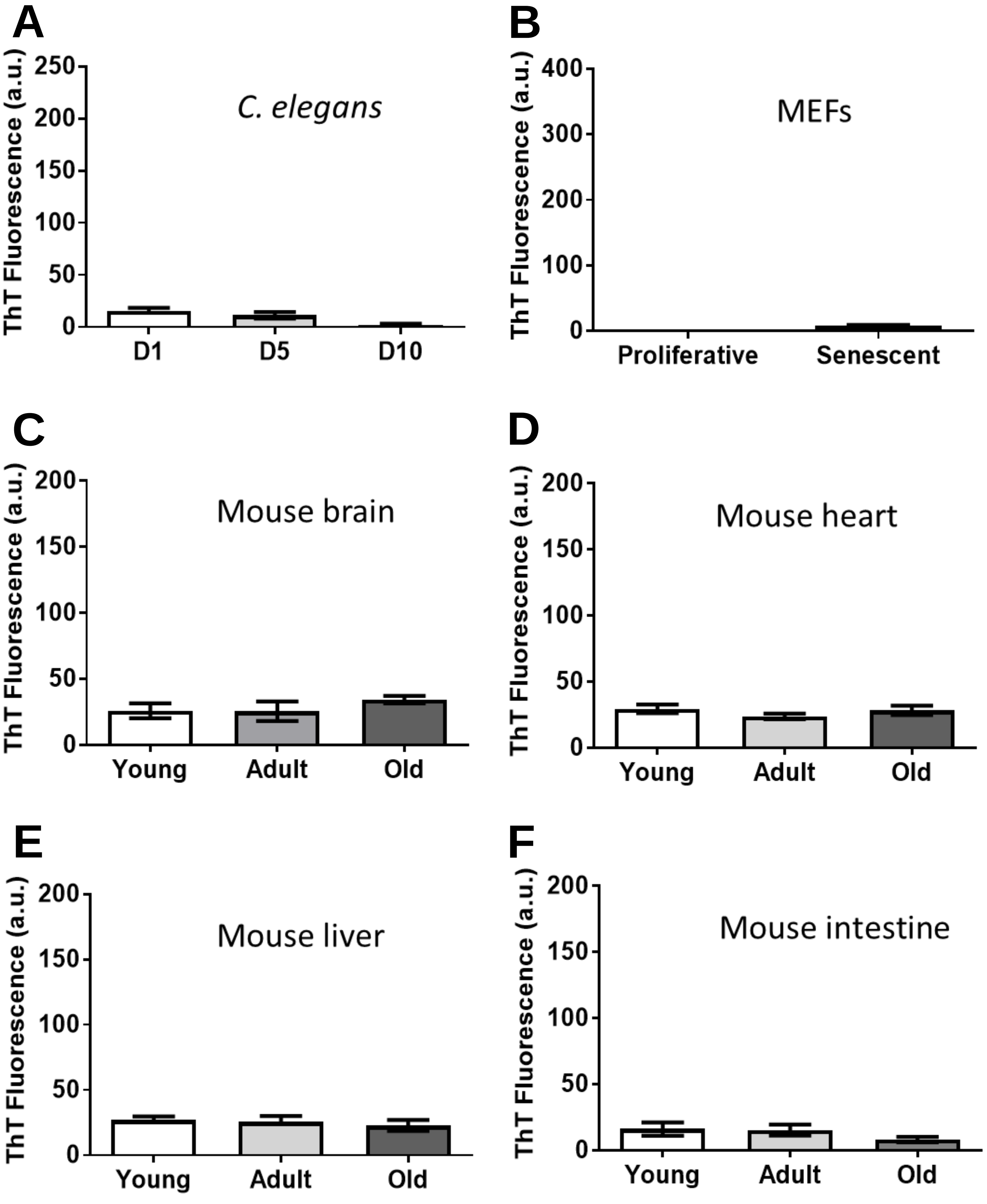

Supplementary Figure 2

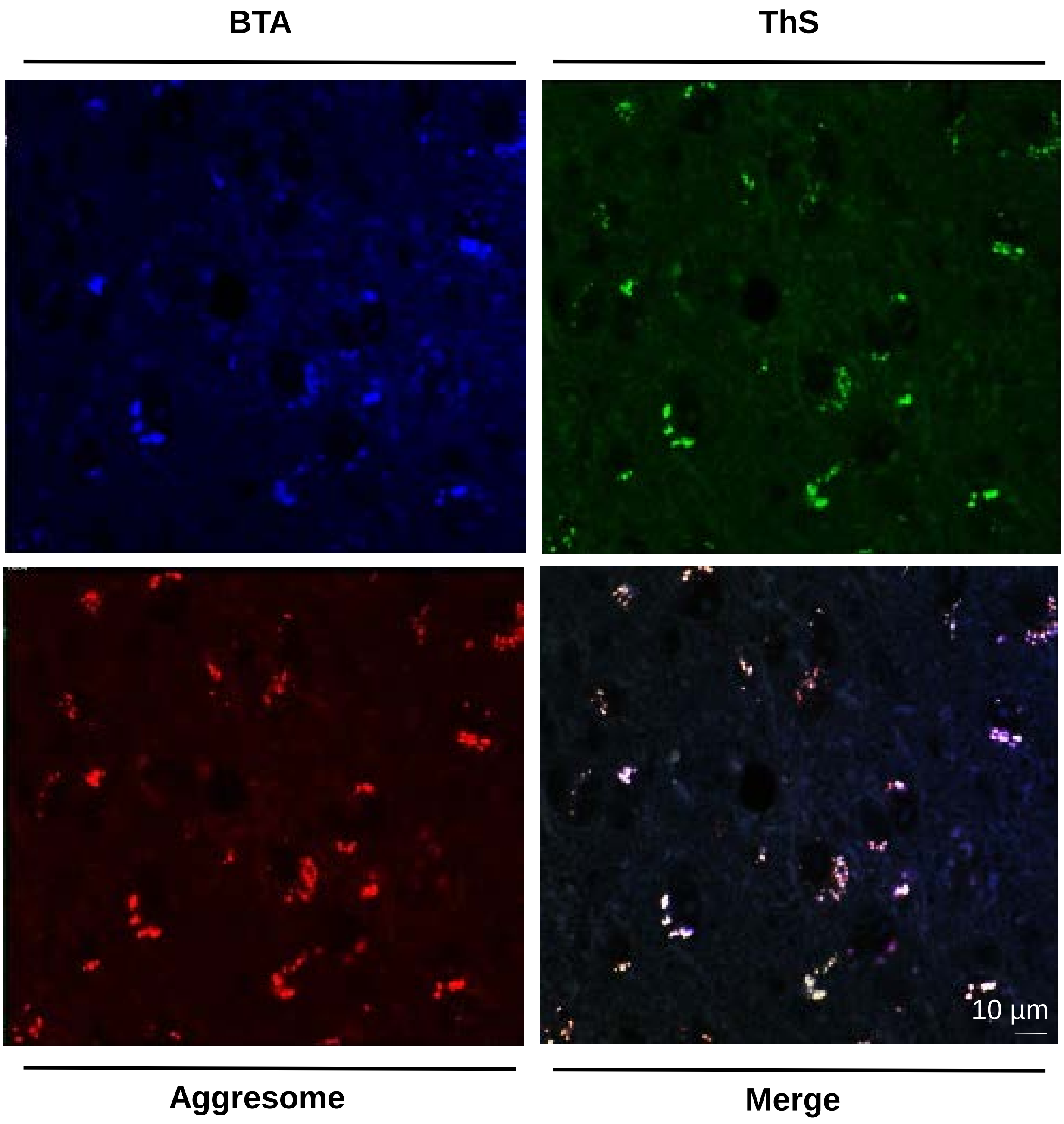

Supplementary Figure 3

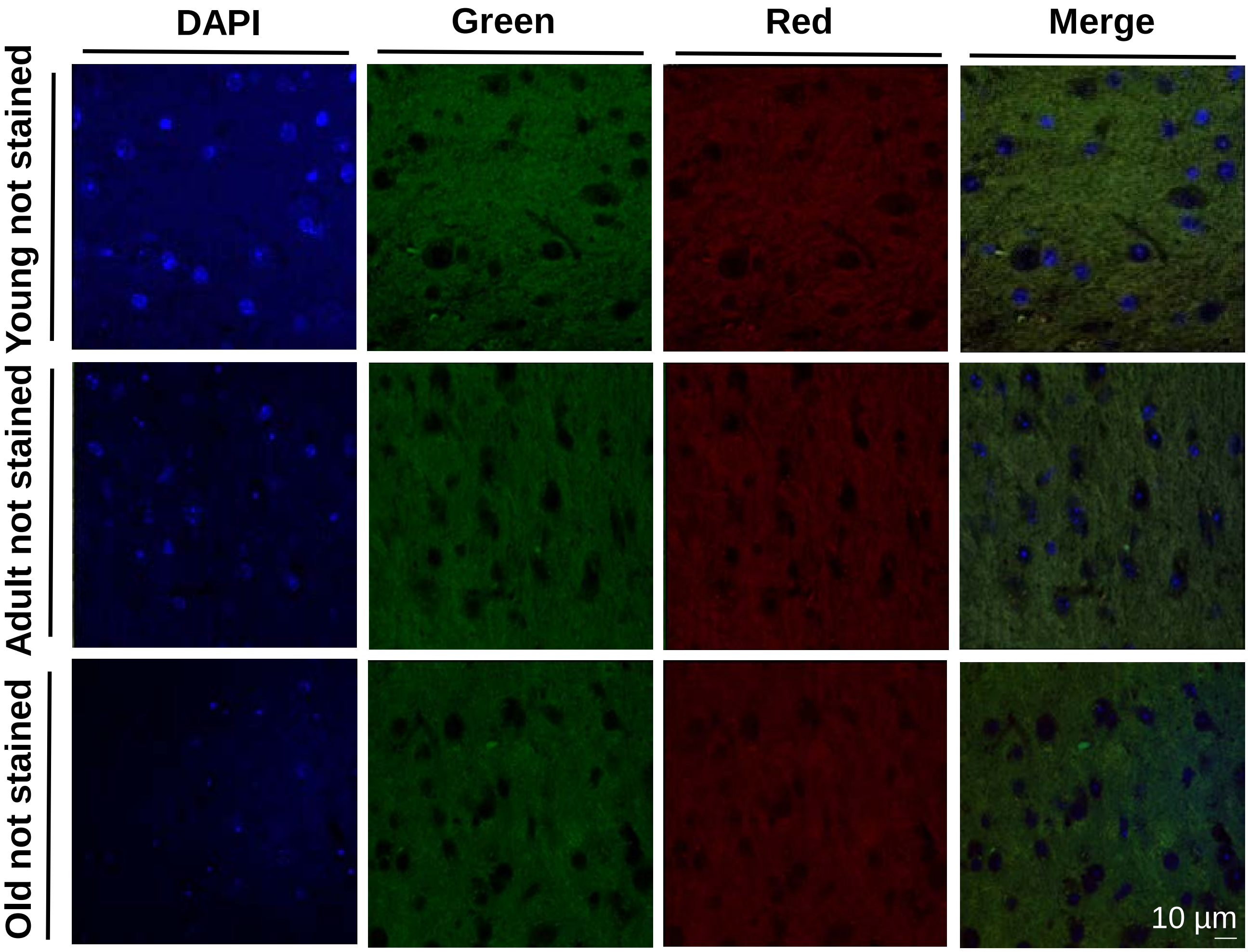

Supplementary Figure 4
